# Evaluation of the biological activities of Copaiba (*Copaifera* spp): a comprehensive review based on scientometric analysis

**DOI:** 10.3389/fphar.2023.1215437

**Published:** 2023-09-01

**Authors:** Deborah Ribeiro Frazão, Jorddy Neves Cruz, Mozaniel Santana de Oliveira, Daiane Claydes Baia-da-Silva, Rayssa Maitê Farias Nazário, Matheus Ferreira de Lima Rodrigues, Miki Taketomi Saito, Renata Duarte Souza-Rodrigues, Rafael Rodrigues Lima

**Affiliations:** ^1^ Laboratory of Functional and Structural Biology, Institute of Biological Sciences, Federal University of Pará, Belém, Pará, Brazil; ^2^ Museu Paraense Emílio Goeldi, Belém, Brazil; ^3^ Faculty of Dentistry, Institute of Health Sciences, Federal University of Pará, Belém, Brazil

**Keywords:** Copaiba oil-resin, biological products, bibliometrics, medicinal plants, traditional medicine

## Abstract

Copaíba oil-resin is extracted from the trunk of the Copaíba tree and has medicinal, cosmetic, and industrial properties. As a result, widespread knowledge about the use of Copaíba oil-resin has evolved, attracting the scientific community’s attention. This paper aims to map the global knowledge production regarding the biological activities of Copaíba (*Copaifera* spp.). Bibliometric methodological instruments were used to conduct a search of the Web of Science-Core Collection database. The search resulted in 822 references. After screening titles and abstracts, 581 references did not meet the eligibility criteria, leaving 246 references for full-text examination. Subsequently, 15 studies were excluded, resulting in a final set of 232 records for the bibliometric analysis. *In vitro* was the most published study type, mainly from Brazil, from 2010 to 2020. Regarding the authors, Bastos, JK, and Ambrosio, SR were the ones with the most significant number of papers included. The most frequent keywords were Copaíba oil, Copaíba, and *Copaifera*. Our findings revealed global study trends about Copaíba, mainly related to its various effects and use over time. In general, all countries have conducted more research on antimicrobial and anti-inflammatory activities, also exposing its antioxidant and healing properties. *Copaifera reticulata* was the most investigated, followed by *Copaifera langsdorffi* and *Copaifera multijuga* in both *in vitro* and *in vivo* studies. Therefore, there is a need for human reports, given the promising results that Copaíba oils have been demonstrating.

## 1 Introduction

**FIGURE 1:**
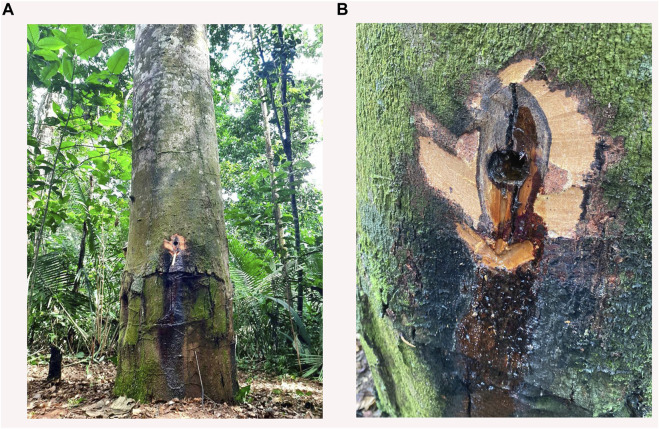
*Copaifera* tree. **(A)** Image of a copaíba tree trunk with **(B)** highlighting the oil-resin collection site. The source of the photo is attributed to the authors.

Humans have used plants to treat several diseases for centuries, but only recently traditional medicine has encouraged novel research with natural products, either bioactive compounds or complex mixtures such as extracts, fixed oils, essential oils, and oil-resin (Petrovska, 2012). Some plant species such as the genus *Copaifera* have different classes of secondary metabolites that may have potential pharmacological applications (Maciel et al., 2002; Palombo, 2011; Mauro et al., 2021). The Copaiba tree (Figure 1) is a member of the Fabaceae family, the Caesalpinioideae subfamily, and the *Copaifera* genus.

Although this genus has several species distributed worldwide (Africa, Central America, and South America), the greatest biodiversity of *Copaifera* is found in Brazil (Arruda et al., 2019). Sixteen out of 28 species of Copaiba cataloged by the Brazilian Agricultural Research Corporation (EMBRAPA) as native from Brazil. These species predominantly grow in the Cerrado and Amazon biomes, which comprise dry and flooded lands, banks of lakes, and streams (Arruda et al., 2019; Dini et al., 2019).

The Brazilian indigenous tribes have benefited from the medicinal properties of *Copaifera* oil-resin since the 16th century (Dini et al., 2019), while this genus was first documented in 1,534 and recorded in the Brazilian Descriptive Pact in 1,587 (Pieri et al., 2009). The pharmacological properties of *Copaifera* oil-resin are attributed to sesquiterpenes and diterpenes components (Cox-Georgian et al., 2019; da Trindade et al., 2018). Although the use of *Copaifera* products decreased in the 18th century, there is a growing demand for herbal medicines and *Copaifera* has gained renewed attention due to potential health benefits (Veiga Junior and Pinto, 2002). This genus has been evaluated by several authors that suggested the oil-resin obtained from these species may benefit human health (Guimarães-Santos et al., 2012; Arruda et al., 2019; Menezes et al., 2022). The Copaiba oil-resin has been used for centuries as a potent analgesic and anti-inflammatory by traditional Amazonians since the 16th century (Dini et al., 2019).

Copaiba has a variety of functions and has proven to be a valuable bioactive, mainly attributed to its anti-inflammatory characteristics (Símaro et al., 2021). It was demonstrated to be twice as effective as diclofenac sodium (Masson et al., 2013), which is the most used anti-inflammatory in the world. Moreover, the oil-resin obtained from the Copaiba tree is a raw material for the European industry, which produces varnishes, perfumes, antitetanics, urinary system antiseptics, antitussives, and cicatrizants. Furthermore, there is a worldwide trend to use Copaiba as biodiesel, which is a less pollutant fuel and preserves the environment. Copaiba oil is an excellent alternative to several medications due to its efficiency, low toxicity, biocompatibility, and low cost (Santos et al., 2011).

Therefore, the widespread knowledge about Copaiba oil-resin has attracted the attention of the scientific community, which progressively seeks answers and solutions to several diseases. The Amazon rainforest’s environmental wealth and size have progressively drawn the world’s attention to what it can offer. A reduced population purchasing power can justify the use of medicinal plants as alternative therapies when drug prices are high and access to medical healthcare is restricted (Battisti et al., 2013). Oils obtained from *Copaifera* species have shown great chemical diversity and potential biological activities (Veiga et al., 2007). Therefore, this study aimed to retrieve the up-to-date published knowledge regarding the biological activities of Copaiba (*Copaifera* spp*.*).

## 2 Methodology

### 2.1 Search method and data source

A bibliometric analysis was used to retrieve global knowledge on the biological activities of Copaiba; thus, articles were searched in the Web of Science Core Collection (WoS-CC), a database that indexes peer-reviewed articles published in high-quality journals and provides a detailed series of accurate information.

To avoid daily update bias, a search was conducted on a single day in June 2022 by two independent examiners by using the following terms: “Copaiba” OR “Copaiba oil” OR “Copaiba oils” OR “Copaiba oil resin” OR “Copaiba oil-resin” OR “Copaiba oleoresin” OR “Copaiba oil resins” OR “Copaiba oil-resins” OR “Copaiba oleoresins” OR “*Copaifer*a” OR “*Copaifera* oil resin” OR “*Copaifera* oil-resin” OR “*Copaifera* oleoresin” OR “*Copaifera* oil resins” OR “Copaifera oil-resins” OR “*Copaifera* oleoresins” or “*Copaifera* species” OR “*Copaifera* spp” OR “*Copaifera* sp” OR “*Copaifera* genus.” Articles were searched from 1945 to 2022 without language restrictions.

### 2.2 Inclusion and exclusion criteria

The inclusion criteria comprised original and complete research articles (*in vitro*, *in situ*, *in vivo*, clinical trials, and narrative/systematic/bibliometric reviews) that investigated oil-resins and essential oil obtained from Copaiba and their biological activities. Letters to the editor and opinion editorials were excluded since they express personal perceptions on a publication or opinions to provide a novel point of view. Moreover, studies that did not primarily investigate the biological activities of Copaiba and studies that exclusively evaluated isolated compounds of Copaiba were excluded.

### 2.3 Article selection

The articles were sorted in descending order of citation number and were independently reviewed by two examiners (DRF and MSO), while a third examiner (RRL) was consulted in case of disagreement.

### 2.4 Data extraction

The following data of the articles were extracted: title, authors, country of origin (based on the corresponding author affiliation), year of publication, number of citations, study design, citation density (number of citations divided by the years since publication), journal, DOI/URL, and keywords. In the case of two articles with identical citation numbers, the article with the highest citation density was upper-ranked.

### 2.5 Data analysis and visualization

Bibliometric networks regarding author co-authorship and author keyword co-occurrences were created by using the Visualization of Similarities Viewer (VOSviewer) software (version 1.6.16) (Center for Science and Technology Studies, University of Leiden, Netherlands) (van Eck and Waltman, 2010). Furthermore, the distribution of articles by continent and country was graphically represented by using the MapChart website (*mapchart.net)*.

### 2.6 Content analysis

The articles were read in full to map all the current knowledge by identifying different species and the biological activity that each publication investigated. All data were manually tabulated on Microsoft Excel In addition, Microsoft Excel, PowerPoint, and Adobe Photoshop were used to rank the most frequent study designs, *Copaifera* species, and biological activities investigated.

## 3 Results

### 3.1 Study selection

**FIGURE 2:**
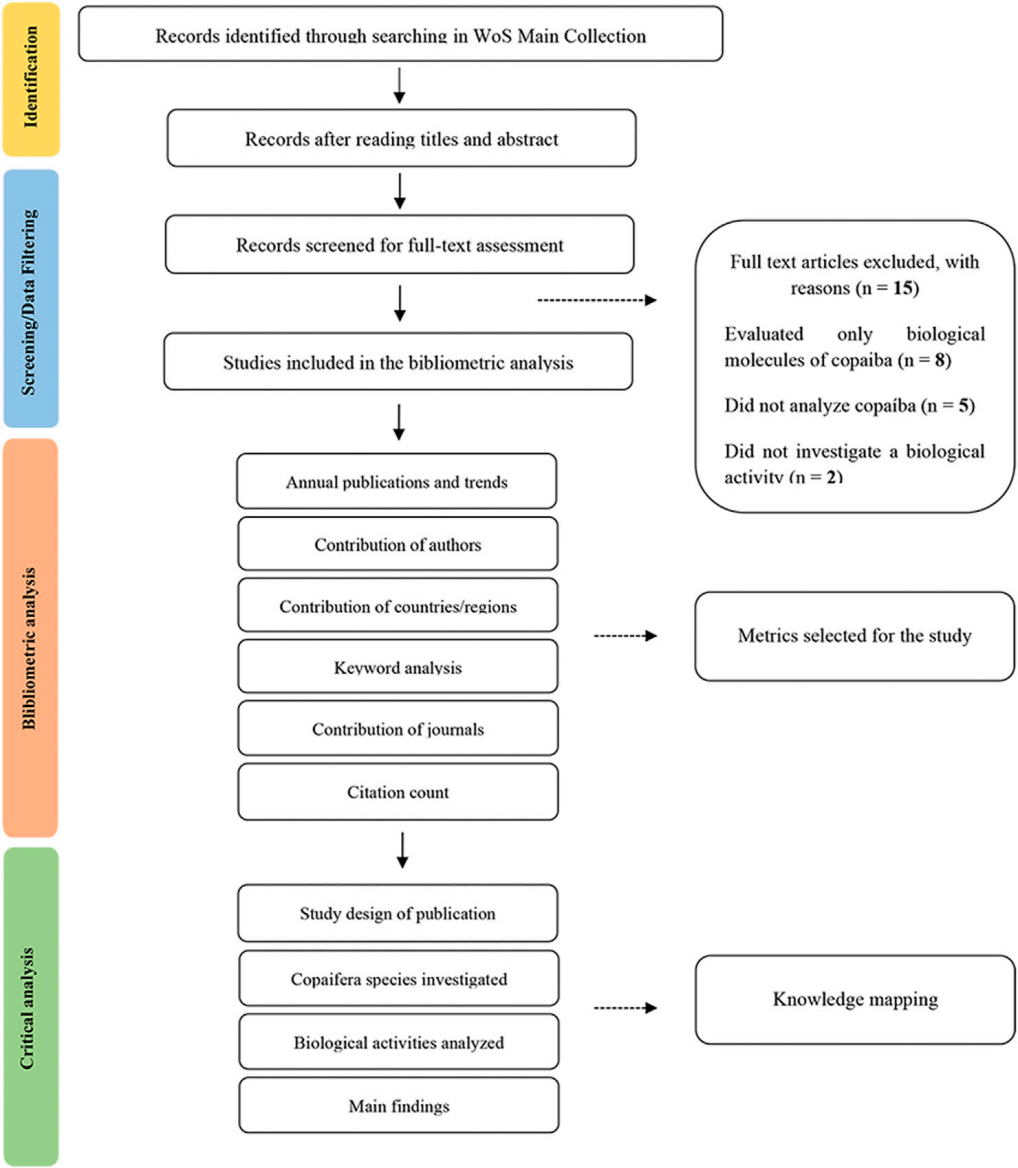
Flow diagram of the study selection process.

After the title and abstract reading, 581 out of 822 references were excluded by following exclusion criteria, and 246 references were selected for full-text reading. Then, 8 studies were excluded since only evaluated isolated compounds of Copaiba essential oil or oil-resin of (Geris et al., 2008; Idippily et al., 2017; Silva et al., 2017; de Carvalho et al., 2018; Pereira et al., 2018; Souza et al., 2018; Farias et al., 2019; Oliveira et al., 2020), 4 studies did not analyze Copaiba (Parreira et al., 2019; Kawakami et al., 2021; do Rosário et al., 2017; Rodrigues da Silva et al., 2020), and 2 articles did not investigate biological activity (Lovelock et al., 1999; Silva et al., 2001). Finally, 232 records were eligible for the bibliometric analysis (Figure 2) and their primary attributes are shown in Supplementary Table S1.

### 3.2 Metrics results

#### 3.2.1 Year of publication

The 232 studies were published between 1983 and 2022. The most productive year in terms of publications was 2020 and 2017, with 24 publications each. The year with the most citations was 2012, with 446 citations, followed by 2007 and 2015, with 373 and 306 citations, respectively. The decade with the most publications (*n* = 142) and citations (*n* = 2,380) was 2010.

Both 2017 and 2020 were the most productive years in terms of number of publications (24 studies each). The highest number of citations (446) was observed in 2012, followed by 2007 and 2015 (373 and 306 citations, respectively). The 2010s was the decade with the most publications (*n* = 142) and citations (*n* = 2,380).

#### 3.2.2 Authors

**FIGURE 3:**
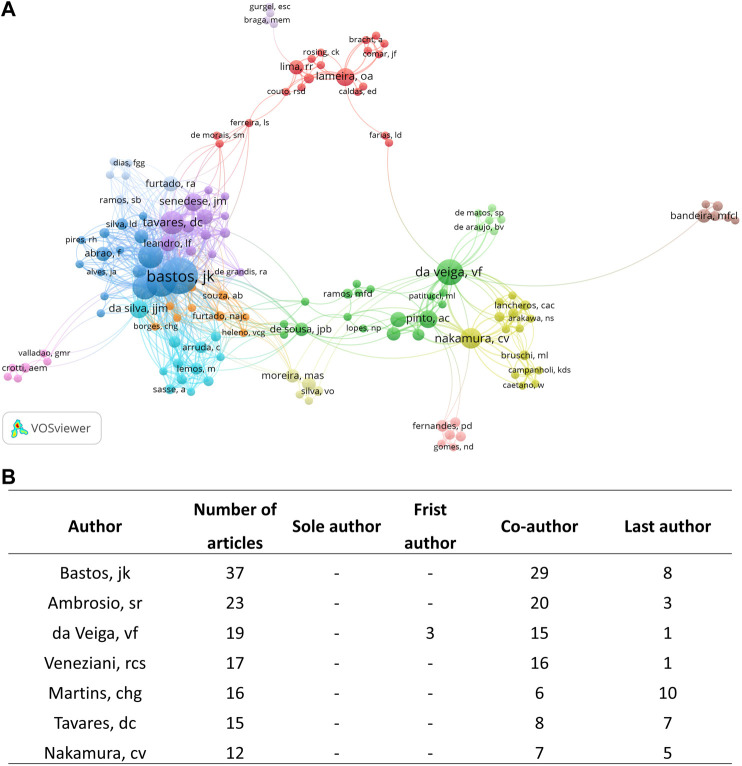
**(A)** Netmap of part of authors’ contribution with at least two published articles. The circles represent the number of articles, and the lines demonstrate the link strength of one author to another. **(B)** Table describing the author’s coauthorship in their manuscripts.

**FIGURE 4:**
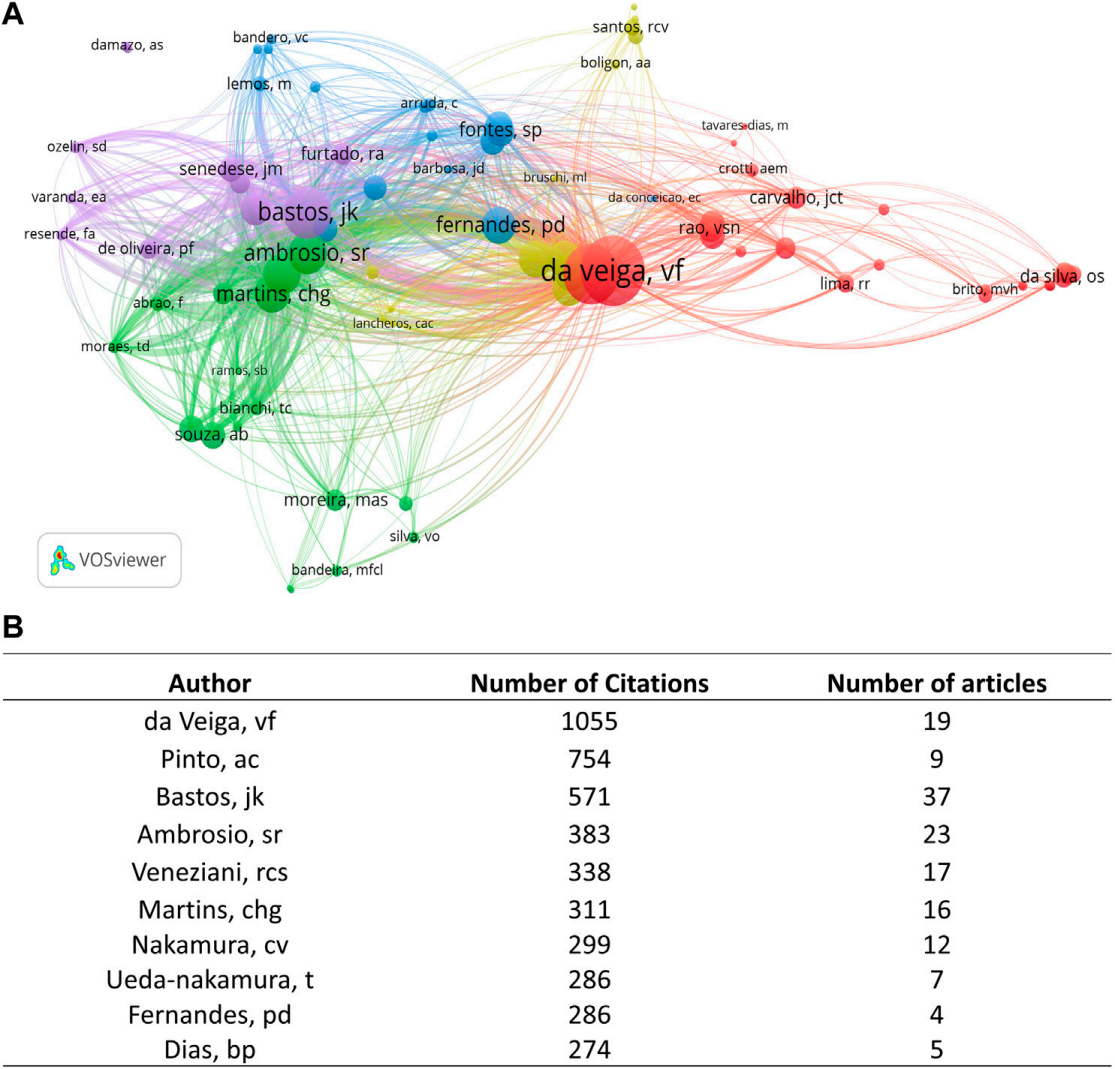
Netmap of part of authors’ citations and integration with other researchers. The circles represent the number of citations, and the lines demonstrate the link strength of one author to another. **(B)** Table describing the Top 10 most cited authors and the number of manuscripts.

Among 1,237 authors, Bastos JK (ORCID iD: 0000–0001–8641–9686; *n* = 37), Ambrosio SR (ORCID iD: 0000–0001–5032–3930; *n* = 23), da Veiga VF (ORCID iD: 0000–0003–1365–7602; *n* = 19), and Veneziani RCS (ORCID iD: Not registered; *n* = 17) contributed to the majority of publications on *Copaifera* spp*.* (Figure 3). The most cited author was Da Veiga VF was the most cited author (1,055 citations), followed by Pinto AC (754 citations), Bastos JK (571 citations), and Ambrosio SR (383 citations) (Figure 4).

#### 3.2.3 Countries

**FIGURE 5:**
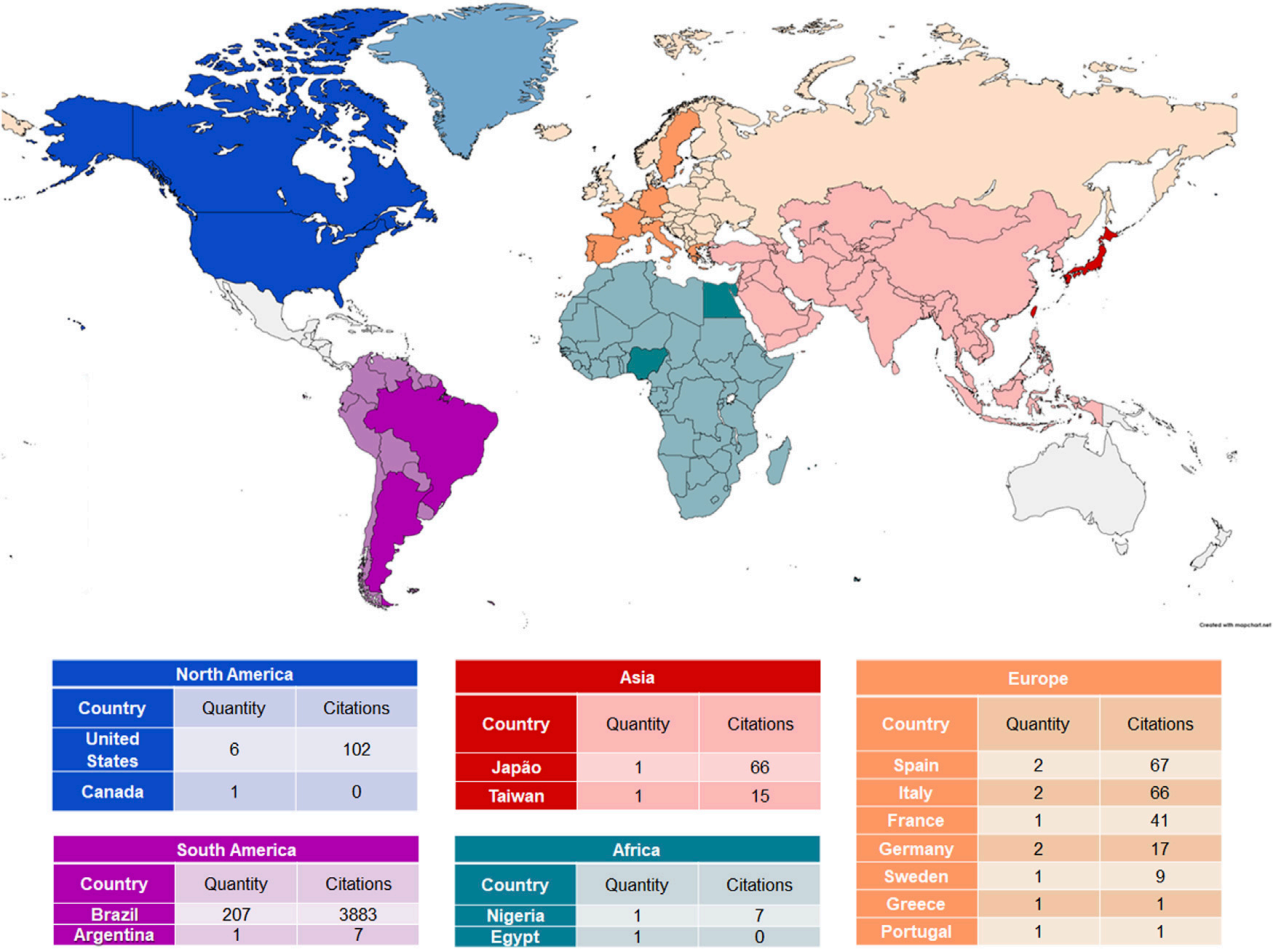
Map of the countries that investigated Copaíba ’s biological activities.

Although the selected articles were published by authors from North America, South America, Europe, Africa, and Asia, only 15 countries of origin were observed: the United States of America (United States), Canada, Brazil, Argentina, Spain, France, Germany, Italy, Sweden, Greece, Portugal, Nigeria, Egypt, Japan, and Taiwan. The highest number of articles originated from Brazil (*n* = 206; 3,883 citations), followed by the United States (*n* = 6; 102 citations), Spain (*n* = 2; 67 citations), and Italy (*n* = 2; 66 citations) (Figure 5).

#### 3.2.4 Keywords

**FIGURE 6:**
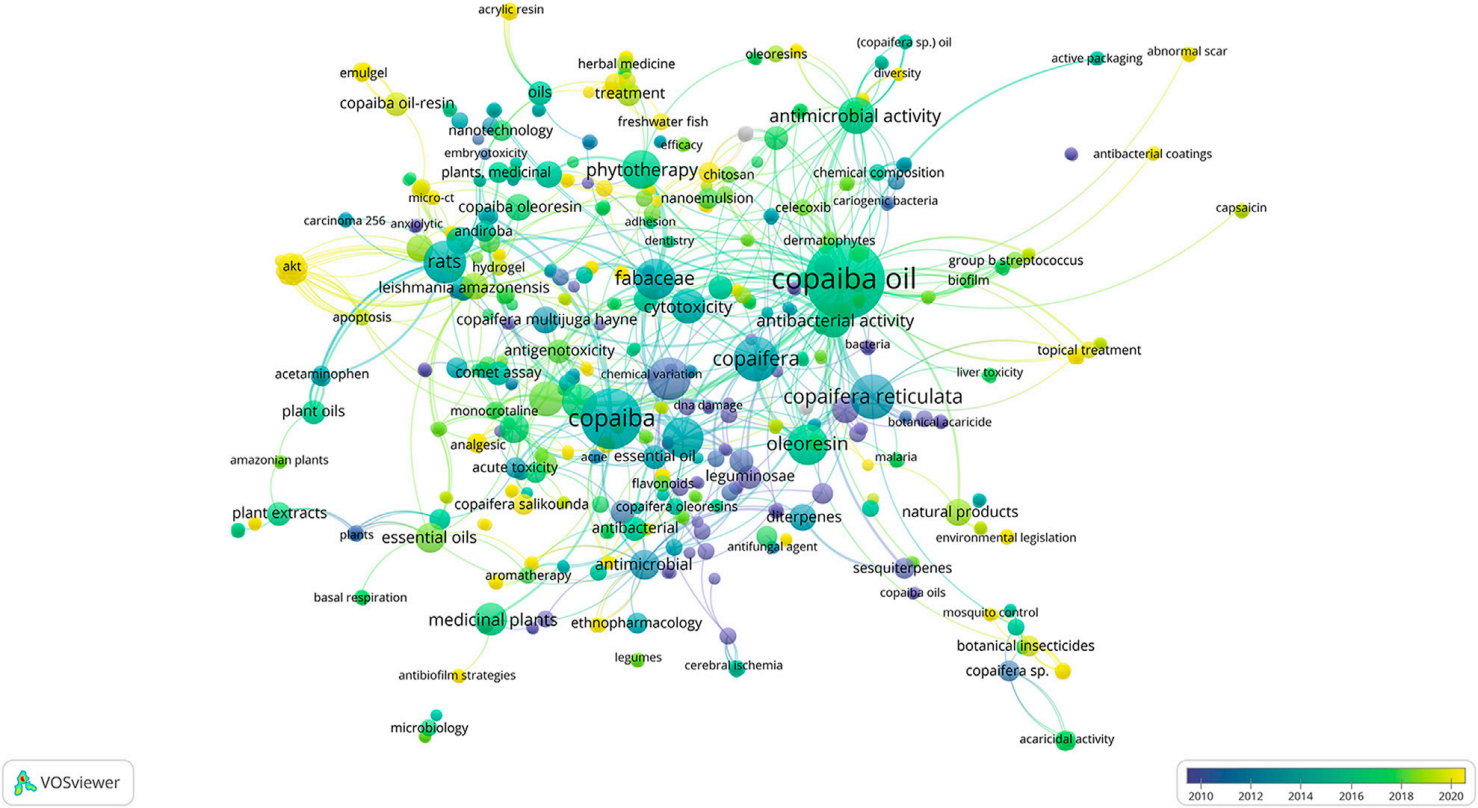
Netmap of keywords found in the included studies organized through the year of use in the last 10 years.

A total of 650 keywords were identified (Figure 6): Copaiba oil (*n* = 40), Copaiba (*n* = 24), *Copaifera* (*n* = 13), *Copaifera reticulata* (*n* = 13), *Copaifera langsdorffii* (*n* = 12), rats (*n* = 12), *Copaifera multijuga* (*n* = 11), Fabaceae (*n* = 11), oil-resin (*n* = 11), phytotherapy (*n* = 10), antimicrobial activity (*n* = 9), and others.

#### 3.2.5 Citation count and citation density

The citation count varied from 0 to 171 (mean 18.53) and the total citation count was 4,289. The review entitled “Medicinal plants: The need for multidisciplinary scientific studies” (Maciel et al., 2002), published in Quimica Nova was the most cited article (171 citations) and its citation density (total citations/mean citations per year) was 8.55. The top 20 most cited articles are shown in Table 1.

**TABLE 1:** Top 20 most cited studies.

Authors (year)	Article title	Times cited, WoS core
MACIEL, MAM; PINTO, AC; VEIGA, VF; GRYNBERG, NF; ECHEVARRIA, A (2002)	Medicinal plants: The need for multidisciplinary scientific studies	171
VEIGA, VF; ROSAS, EC; CARVALHO, MV; HENRIQUES, MGMO; PINTO, AC (2007)	Chemical composition and anti-inflammatory activity of copaiba oils from *Copaifera cearensis* Huber ex Ducke, *Copaifera reticulata* Ducke and *Copaifera multijuga* Hayne - A comparative study	165
LEANDRO, LM; VARGAS, FD; BARBOSA, PCS; NEVES, JKO; DA SILVA, JA; DA VEIGA, VF (2012)	Chemistry and Biological Activities of Terpenoids from Copaiba (*Copaifera* spp.) Oleoresins	150
DOS SANTOS, AO; UEDA-NAKAMURA, T; DIAS, BP; VEIGA, VF; PINTO, AC; NAKAMURA, CV (2008)	Antimicrobial activity of Brazilian copaiba oils obtained from different species of the *Copaifera* genus	109
GOMES, NM; REZENDE, CM; FONTES, SP; MATHEUS, ME; FERNANDES, PD (2007)	Antinociceptive activity of Amazonian Copaiba oils	107
SANTOS, AO; UEDA-NAKAMURA, T; DIAS, BP; VEIGA, VF; PINTO, AC; NAKAMURA, CV (2008)	Effect of Brazilian copaiba oils on *Leishmania amazonensis*	99
ANDRADE, BFMT; BARBOSA, LN; PROBST, ID; FERNANDES, A (2014)	Antimicrobial activity of essential oils	91
LIMA, SRM; VEIGA, VF; CHRISTO, HB; PINTO, AC; FERNANDES, PD (2003)	*In vivo* and *in vitro* studies on the anticancer activity of *Copaifera* multijuga Hayne and its fractions	88
VEIGA, VF; ZUNINO, L; CALIXTO, JB; PATITUCCI, ML; PINTO, AC (2001)	Phytochemical and antioedematogenic studies of commercial copaiba oils available in Brazil	84
DE MENDONCA, FAC; DA SILVA, KFS; DOS SANTOS, KK; JUNIOR, KALR; SANT'ANA, AEG (2005)	Activities of some Brazilian plants against larvae of the mosquito *Aedes aegypti*	83
SOUZA, AB; MARTINS, CHG; SOUZA, MGM; FURTADO, NAJC; HELENO, VCG; DE SOUSA, JPB; ROCHA, EMP; BASTOS, JK; CUNHA, WR; VENEZIANI, RCS; AMBROSIO, SR (2011)	Antimicrobial Activity of Terpenoids from *Copaifera* langsdorffii Desf. Against Cariogenic Bacteria	82
FERNANDES, FD; FREITAS, EDP (2007)	Acaricidal activity of an oleoresinous extract from *Copaifera* reticulata (Leguminosae: Caesalpinioideae) against larvae of the southern cattle tick, *Rhipicephalus* (*Boophilus*) *microplus* (Acari: Ixodidae)	80
BASILE, AC; SERTIE, JAA; FREITAS, PCD; ZANINI, AC (1988)	Anti-Inflammatory Activity of Oleoresin from Brazilian *Copaifera*	75
DE LIMA, MRF; LUNA, JD; DOS SANTOS, AF; DE ANDRADE, MCC; SANT'ANA, AEG; GENET, JP; MARQUEZ, B; NEUVILLE, L; MOREAU, N (2006)	Anti-bacterial activity of some Brazilian medicinal plants	75
PAIVA, LAF; RAO, VSN; GRAMOSA, NV; SILVEIRA, ER (1998)	Gastroprotective effect of *Copaifera langsdorffii* oleo-resin on experimental gastric ulcer models in rats	74
SOUZA, AB; DE SOUZA, MGM; MOREIRA, MA; MOREIRA, MR; FURTADO, NAJC; MARTINS, CHG; BASTOS, JK; DOS SANTOS, RA; HELENO, VCG; AMBROSIO, SR; VENEZIANI, RCS (2011)	Antimicrobial Evaluation of Diterpenes from *Copaifera langsdorffii* Oleoresin Against Periodontal Anaerobic Bacteria	72
POHLIT, AM; REZENDE, AR; BALDIN, ELL; LOPES, NP; NETO, VFD (2011)	Plant Extracts, Isolated Phytochemicals, and Plant-Derived Agents Which Are Lethal to Arthropod Vectors of Human Tropical Diseases - A Review	66
OHSAKI, A; YAN, LT; ITO, S; EDATSUGI, H; IWATA, D; KOMODA, Y (1994)	The Isolation and *in vivo* Potent Antitumor-Activity of Clerodane Diterpenoid from the Oleoresin of the Brazilian Medicinal Plant, *Copaifera l*angsdorfii Desf	66
TINCUSI, BM; JIMENEZ, IA; BAZZOCCHI, IL; MOUJIR, LM; MAMANI, ZA; BARROSO, JP; RAVELO, AG; HERNANDEZ, BV (2002)	Antimicrobial terpenoids from the oleoresin of the Peruvian medicinal plant *Copaifera* paupera	64
BONAN, RF; BONAN, PRF; BATISTA, AUD; SAMPAIO, FC; ALBUQUERQUE, AJR; MORAES, MCB; MATTOSO, LHC; GLENN, GM; MEDEIROS, ES; OLIVEIRA, JE (2015)	*In vitro* antimicrobial activity of solution blow spun poly (lactic acid)/polyvinylpyrrolidone nanofibers loaded with Copaiba (*Copaifera* sp.) oil	61

#### 3.2.6 Journal ranking

**FIGURE 7:**
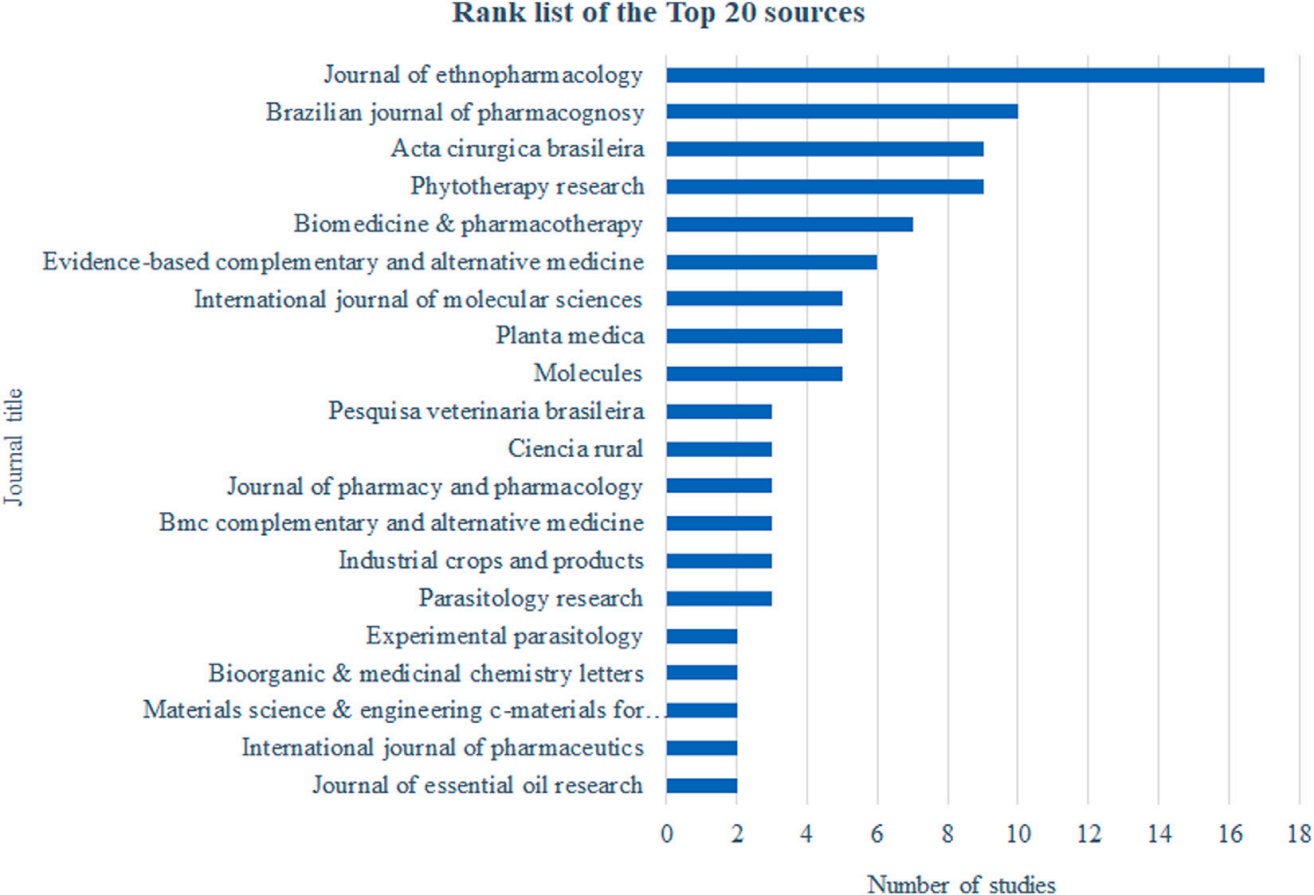
Journal rankings of Copaíba’s biological activities knowledge production.

The highest number of articles on the biological activities of Copaiba was published by the Journal of Ethnopharmacology (*n* = 16), followed by the Brazilian Journal of Pharmacognosy (*n* = 10). The top 20 journals in terms of number of published articles are shown in Figure 7.

### 3.3 Content results

#### 3.3.1 Study design

**FIGURE 8:**
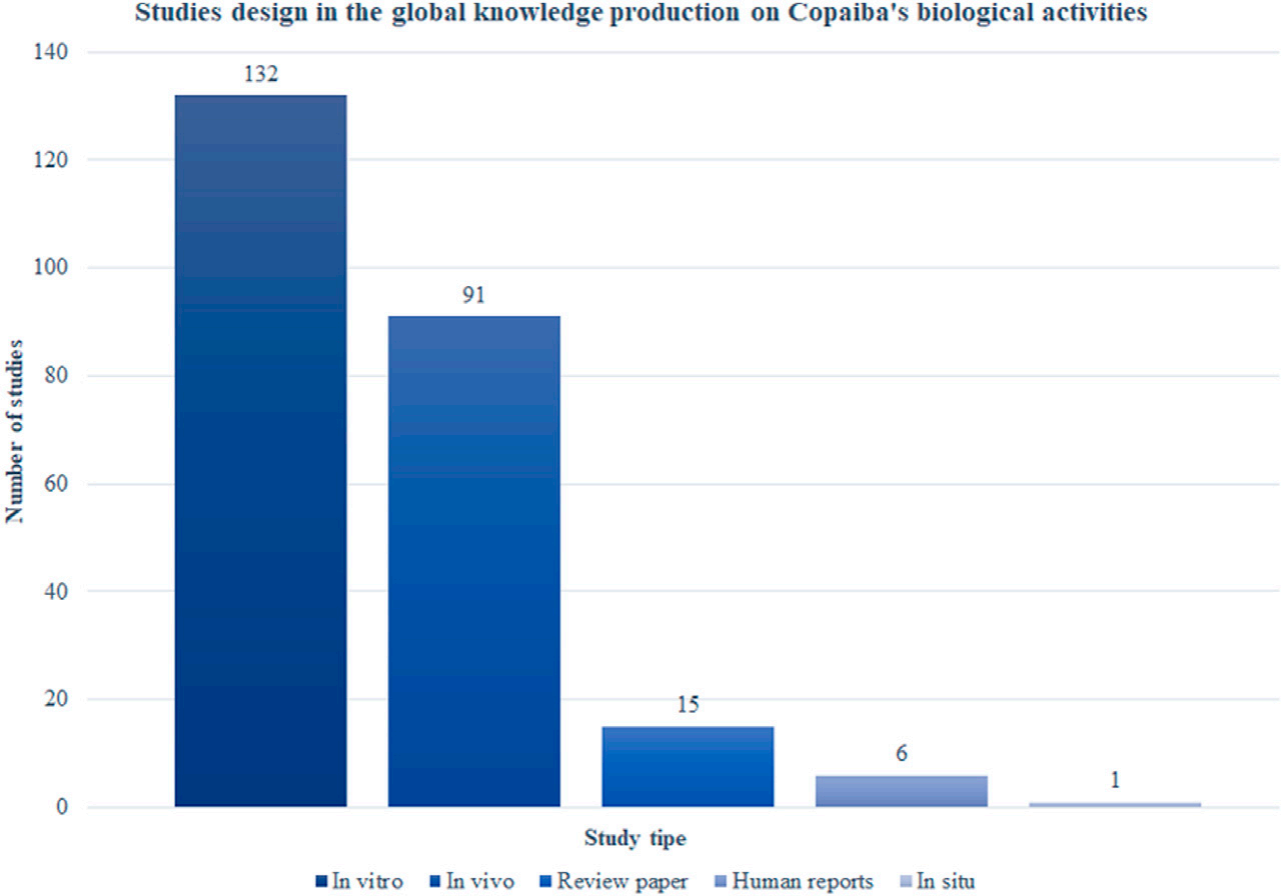
Ranking of study types on the global knowledge production on Copaíba ’s biological activities.

The majority of studies on the biological activities of Copaiba were *in vitro* (*n* = 132; 59.70%), followed by *in vivo* (*n* = 91; 39.39%), review articles (*n* = 15; 6.49%), and clinical trials (*n* = 6; 2.59%) (Figure 8). It must be addressed that 17 studies combined *in vivo* and *in vitro* experiments.

#### 3.3.2 *Copaifera* species

**FIGURE 9:**
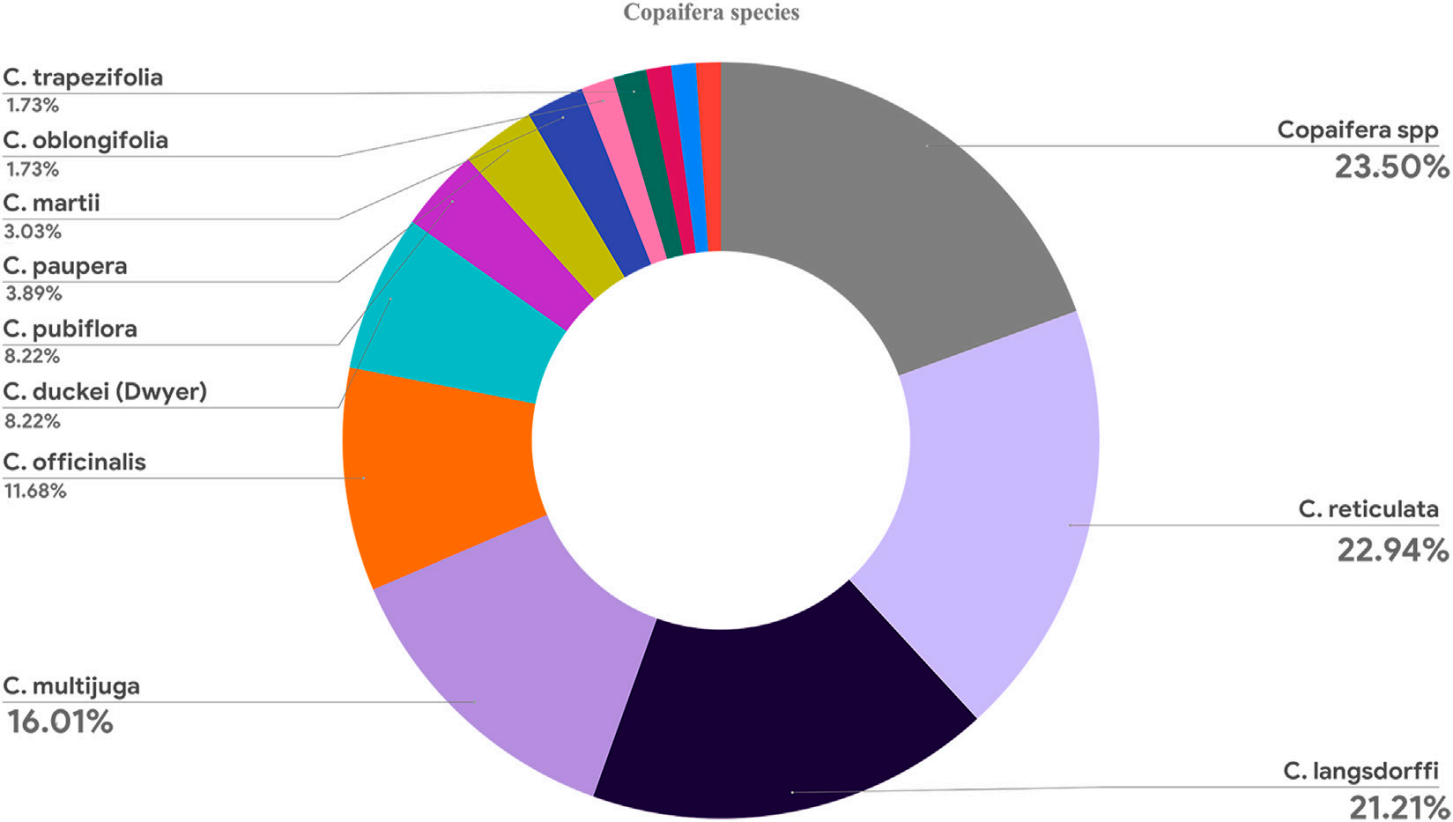
Graphic showing all *Copaifera* species investigated in the articles.

Although 12 *Copaifera* spp. Species were described in the articles, most papers reviewed multiple species or did not detail them (*n* = 55). Nevertheless, *C. reticulata* (*n* = 53), *Copaifera langsdorffi* (*n* = 48), *C. multijuga* (*n* = 37), and *Copaifera officinalis* (*n* = 27) were the most investigated species. Figure 9 shows that other species were found in less than one-tenth of the articles.

#### 3.3.3 Biological activities

**FIGURE 10:**
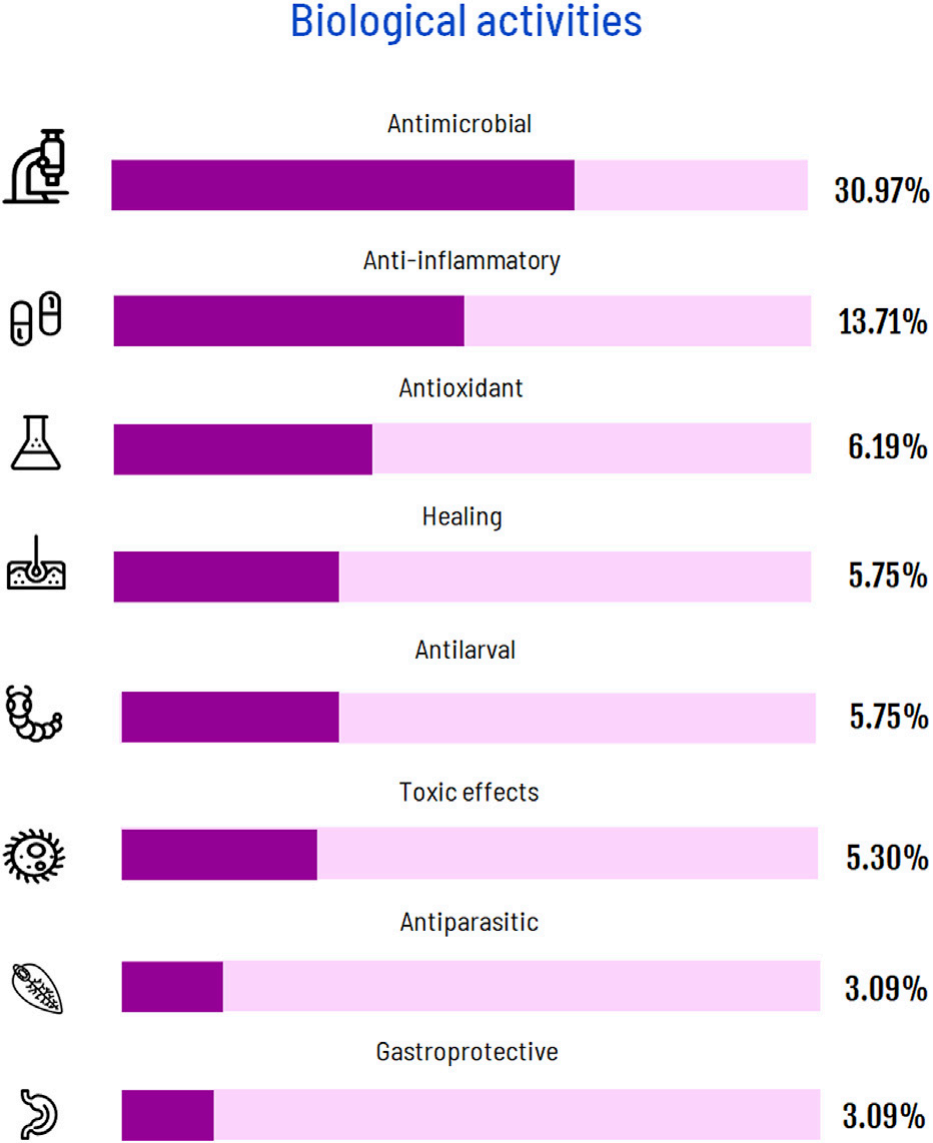
Graphic showing all biological activities investigated in the articles.

The articles revealed 13 biological activities of Copaiba: antimicrobial (antibacterial and antifungal) (30%), anti-inflammatory (13.71%), antioxidant (6.19%), healing/cicatrization (5.75%), antilarval (5.75%), antiparasitic (4.42%), gastroprotective (3.09%), insecticidal (1.76%), anticancer/anti-tumor (1.76%), and antinociceptive (1.76%) (Figure 10). Surprisingly, toxicological studies (5.3%) revealed that Copaiba does not harm human organisms.

## 4 Discussion

This literature review was based on a bibliometric analysis method, which addressed the global knowledge regarding the biological activities of Copaiba. Although most articles did not detail the investigated species, *C. reticulata* was the most frequently reported. In addition, *in vivo* and antimicrobial were respectively the most common study design and biological activity studied. Although Copaiba has been investigated since only the 1980s, this relatively recent topic was investigated by a great number of authors from several countries from almost all continents.

More than 11 Copaiba species were detailed in the selected studies. Furthermore, some studies investigated more than one species since their phytochemical profile changes in accordance with region, time, plant part, and detection method (Arruda et al., 2019). Nevertheless, Copaiba oil-resins and essential oils contain common components responsible for biological activities.

**FIGURE 11:**
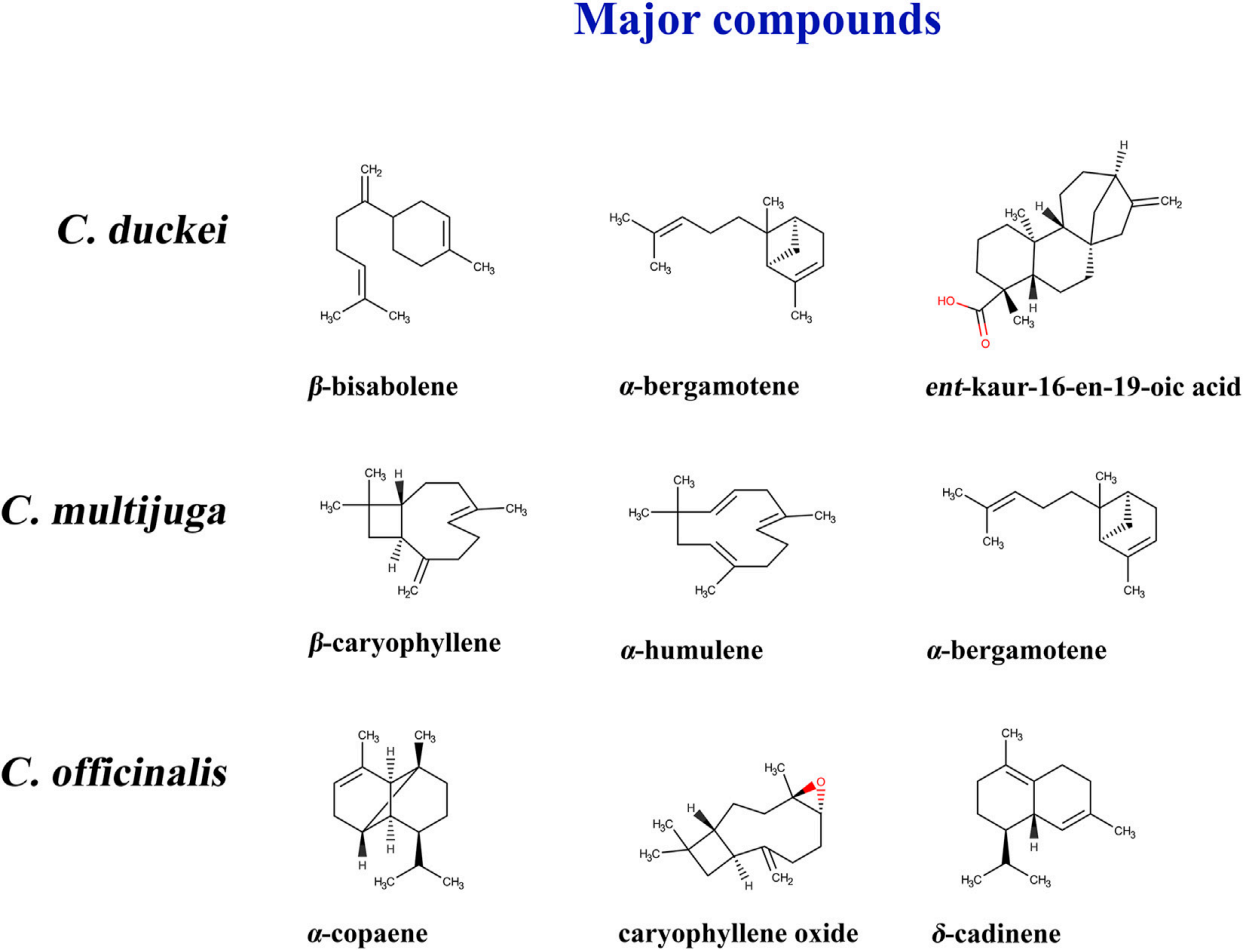
Compounds found in some copaiba species.

The literature indicates two major groups of components: sesquiterpenes and diterpenes (Figure 11). Terpenes are secondary metabolites with varied molecular structures that play a key role in essential sensory characteristics such as smell, taste, and color. β-Caryophyllene, trans-α-bergamotene, and β-bisabolene are sesquiterpenes present in Copaiba oil-resin. β-Caryophyllene is known as a significant marker of Copaiba oil-resin obtained from some species (Abrão et al., 2018). Copalic acid, polyalthic acid, and kaurenoic acid are diterpenes found in several species such as *C. paupera* (Herzog) Dwyer and *C. reticulata Ducke* (Lameira et al., 2009; Guimarães-Santos et al., 2012). The literature demonstrates that a wide range of biological processes can be attributed to these components found in both oil and extracts derived from different plant parts.

Most of the oils obtained from *Copaifera duckei* contain β-bisabolene, α-bergamotene, and kaur-16-en19-oic, and their biological activity is usually associated with the synergistic effect of all compounds. β-Bisabolene can be used as a flavoring agent (Dionisio et al., 2018) and presents a high antioxidant capacity (up to 14 ± 0.8 mg/mL in accordance with the DPHH method). Even though this major compound is mixed with other chemicals for essential oil production, β-bisabolene has antimicrobial effects against Gram-positive and Gram-negative bacteria (Dionisio et al., 2018). α-Bergamotene is a volatile substance combined with other compounds in several essential oils and is related to antioxidant capacity without toxic effects (Moraes et al., 2022). Kaur-16-en19-oic has antimicrobial activity against Gram-positive bacteria (Vieira et al., 2002).

The oil obtained from *C. multijuga* contains β-caryophyllene, α-humulene, and α-bergamotene as major components. β-Caryophyllene is a food additive that can modulate inflammatory processes. It can be consumed alone or contained in certain edible plant species such as black pepper (Gertsch et al., 2008). Topical application or oral administration of α-humulene can reduce inflammation and pain as demonstrated in an *in vivo* study with rats (Chaves et al., 2008). α-Copaene, which is one of the primary compounds of oils obtained from *C. officinalis*, is associated with antioxidant properties and did not show cytotoxicity through the 3-(4,5-dimethylthiazol-2-yl)-2,5-diphenlytetrazolium bromide test (Turkez et al., 2014).

These several compounds found in Copaiba have come to the attention of researchers from medicine, dentistry, and other fields. Sixteen of 77 medical studies indicated promising effects of *C. langsdorffi* against several diseases or conditions such as Alzheimer’s (Penido et al., 2017)–47], urolithiasis (Brancalion et al., 2012), acne (da Silva et al., 2012), microorganism infections (Tincusi et al., 2002; de Lima et al., 2006; Tobouti et al., 2017), cancer (Abrão et al., 2015; Senedese et al., 2019), intestinal injury (Paiva et al., 1998; Adzu et al., 2015). Nevertheless, the first medical study investigated the anti-inflammatory effect of *C. reticulata Ducke* on carrageenin-induced pedal edema in rats (Basile et al., 1988). This model was widely used between 1960 and 1980 to investigate the anti-inflammatory potential of natural products such as African spices (Fernández-Moriano et al., 2019).

Moreover, *in vivo* (mainly animal models) was the most common type of medical study (*n* = 36). It is also important to mention that 3 out of 6 clinical trials found in this review originated from the medical field (da Silva et al., 2012; Bahr et al., 2018; Waibel et al., 2021). These trials have a considerably higher certainty of evidence than *in vitro* and *in vivo* studies; however, a low number of clinical trials were published (Murad et al., 2016). The placebo-controlled clinical trial conducted in 2012 showed that Copaiba essential oil reduced the face area affected by acne (da Silva et al., 2012). In 2018, another clinical trial showed the analgesic and anti-inflammatory effects of Copaiba essential oil used to hand massage individuals with arthritis and osteoarthritis (Bahr et al., 2018). Recently in 2021, a prospective, randomized, double-blind, and placebo-controlled clinical trial showed that the use of a Copaiba oil-containing silicone-based gel for 84 days improved the color, contour, distortion, and texture of different types of scars (Vasconcelos et al., 2008).

The number of studies in the oral science field has been constantly growing due to the potential antimicrobial effect of Copaiba, especially against oral pathogens. Although the first investigation of Copaiba in the dental field was published in the 2000s (Bandeira et al., 2000), this subject was highlighted in the scientific community only in the 2010s due to the need for safe, effective, and low-cost alternative methods to prevent and treat diseases (Palombo, 2011). Therefore, 17 out of the 25 studies (68%) on the use of Copaiba in the oral cavity and orofacial region were published between 2010 and 2020.

In the 2000s, the increase in the number of oral diseases, financial issues in underdeveloped countries, and bacteria resistance to regular antibiotics intensified research on adjuvant treatments such as phytotherapeutic compounds that could treat severe infections caused by multiresistant bacteria (Palombo, 2011). Phytomedicine has been used in dentistry as an anti-inflammatory, antibacterial, analgesic, sedative, and endodontic irrigant (Groppo et al., 2008). Copaiba’s anti-inflammatory and antimicrobial effects observed in the medical field suggested its use on the oral cavity.

Therefore, several studies investigated the effect of Copaiba on endodontic materials (Bandeira et al., 2000; Vasconcelos et al., 2008; Couto et al., 2020; Reiznautt et al., 2021), periodontal anaerobic bacteria (Souza et al., 2011a), periodontitis (Ohsaki et al., 1994), antimicrobial effect on plaque (Pieri et al., 2010; Pieri et al., 2013), cariogenic (Souza et al., 2011b; Moraes et al., 2016; Moraes et al., 2020; dos Santos et al., 2021), and anti-inflammatory and healing effects on tongue lesion (Teixeira et al., 2017; Wagner et al., 2017; Alvarenga et al., 2020). One study reported the lack of the Copaiba effect on mandibular bone, which could considerably benefit buccomaxillofacial surgery and orthodontics (Silva et al., 2015). The unique clinical trial that evaluated the effects of a Copaiba-containing varnish on children’s teeth to decrease the risk of dental caries (Rocha Valadas et al., 2021). Moreover, the unique *in situ* study found in this bibliometric analysis originated from the dentistry field and evaluated the antiproteolytic activity of Copaiba oil-based emulsion at the resin/dentin adhesive interface (Araújo et al., 2021).

A global distribution analysis showed that most studies were conducted in Brazil, which is the country that presents the most significant number of species worldwide. The Brazilian climate, particularly in the Amazon region, favors the growth of Copaiba trees. The hot and humid Amazon biome has abundant rainfall, a diverse range of ecosystems, and represents 49% of the Brazilian territory (Moraes et al., 2020; dos Santos et al., 2021). Nevertheless, the first research on Copaiba was conducted in 1983 at the University of California (United States) and published in Biochemistry and Molecular Biology (Arrhenius and Langenheim, 1983). Then in 1988, researchers from the University of São Paulo (Brazil) showed that dose-dependent administration of Copaiba oil-resin prevented the development of carrageenin-induced pedal edema in female rats; in addition, repeated administration of the oil-resin at 1.26 mL/kg for 6 days considerably reduced the permeability increase caused by histamine (Basile et al., 1988). Despite the promising results, the second Brazilian article on Copaiba was published only 10 years later and showed that oral administration of the oil-resin obtained from *C. langsdorffii* at 200 and 400 mg/kg provided dose-dependent protection against ethanol-induced gastric damage. Moreover, the administration of *C. langsdorffii* oil-resin at 400 mg/kg protected against indomethacin-induced gastric ulceration. In addition, the oil-resin significantly increased the accumulation of gastric juice volume and mucus secretion and suppressed overall acidity in pylorus-ligated rats after 4 h (Paiva et al., 1998).

In the meantime, a Japanese research investigated the anti-tumor potential of *C. langsdorffii* and its compounds (particularly the diterpenoid clerodane) (Ohsaki et al., 1994), while another *in vitro* study in Argentina demonstrated that *C. reticulata* inhibited free radical-mediated DNA damage (Desmarchelier et al., 1997). In 2002, a study originated from Spain determined the significant leishmanicidal, antimicrobial, cytotoxic, and aldose reductase inhibitory activities of compounds isolated from *C. paupera* (Senedese et al., 2019).

Henceforth, Brazil stood out in the research on biological activities of Copaiba for 10 years through the publication of *in vitro* studies as well as *in vivo* studies with animals. Furthermore, the first clinical trial on the effects of Copaiba against acne was conducted. Between 2003 and 2013, there was also an increase in the number of studies on antimicrobial activities, including bacteria, fungi, and viruses.

The studies usually investigate antibacterial activity through the minimum inhibitory concentration (MIC) method, which determines the *in vitro* sensitivity or resistance of specific bacterial strains to an antibiotic. The MIC of an antibiotic is the lowest concentration at which the growth of a particular strain of bacteria is completely inhibited under strictly controlled conditions. Recently, this method has been routinely reported for standard testing (Desmarchelier et al., 1997).

This bibliometric analysis found that most of the studies were conducted *in vitro* and very few clinical trials were published. Although *in vitro* and *in vivo* studies are needed, the level of evidence of the effects of Copaiba must be validated by studies with humans; in addition, there is a lack of either *in vivo* and *in vitro* investigations on mineralized tissues. Furthermore, most studies did not detail the species of Copaiba, probably due to the use of a commercial sample. However, some variations in the compounds among species may alter the biological activities induced in the same target.

## 5 Conclusion

This bibliometric analysis explored the global knowledge developed over the years regarding the biological activities of Copaiba. The comprehensive evaluation of 11 species was mainly based on *in vivo* investigations that emphasized antimicrobial activity. There is a growing interest in Copaiba within medical and dental fields, in which numerous studies explored potential applications across several specialties. Nevertheless, further research is required to validate the effects of Copaiba in humans, as well as the comparison among different Copaiba species. Overall, this review highlights the extensive potential of Copaiba and the importance of advanced research to support its evidence-based use.

## Data Availability

The original contributions presented in the study are included in the article/Supplementary Material, further inquiries can be directed to the corresponding author.
