# Supplementary material for: Evaluation of the biological activities of Copaiba (Copaifera spp): a comprehensive review based on scientometric analysis
Source: Front Pharmacol. 2023 Sep 1;14:1215437. doi: 10.3389/fphar.2023.1215437 (PMC10502340; doi:10.3389/fphar.2023.1215437)
Supplement: Supplementary file 1 [file Table1.DOCX]

Supplementary Material

Scientific production evaluation about Copaiba’s (*Copaifera* spp) biological activities: a global and historical knowledge mapping based on scientometric analysis

**Deborah Ribeiro Frazão^1^, Jorddy Neves Cruz^1^, Mozaniel Santana de Oliveira^2^, Daiane Claydes Baia-da-Silva^1^, Rayssa Maitê Farias Nazário^1^, Matheus Ferreira de Lima Rodrigues^1^, Miki Taketomi Saito^3^, Renata Duarte Souza-Rodrigues^1^, Rafael Rodrigues Lima^1,^***

*** Correspondence:**Ph.D. Rafael Rodrigues Lima.
[rafalima@ufpa.br](mailto:rafalima@ufpa.br)

Supplementary Table 1: Summary of the manuscripts on Copaiba's biological activity in the Web of Science Core Collection: Comprehensive Data Overview.

| Authors | Year | Article title | *Copaifera* species | Biological activities | Study design | Journal of publication | Times Cited, wos Core | Citation Density | DOI |
| --- | --- | --- | --- | --- | --- | --- | --- | --- | --- |
| ARRHENIUS, SP; LANGENHEIM, JH | 1983 | Inhibitory effects of hymenaea and copaifera leaf resins on the leaf fungus, pestalotia-subcuticularis | *Copaifera multijuga* | Antifungal | In vitro | Biochemical systematics and ecology | 39 | 1 | 10.1016/0305-1978(83)90037-6 |
| BASILE, AC; SERTIE, JAA; FREITAS, PCD; ZANINI, AC | 1988 | Anti-inflammatory activity of oleoresin from brazilian copaifera | *Copaifem reticulata Ducke* | Anti-inflammatory | In vivo | Journal of ethnopharmacology | 75 | 2,20 | 10.1016/0378-8741(88)90235-8 |
| OHSAKI, A; YAN, LT; ITO, S; EDATSUGI, H; IWATA, D; KOMODA, Y | 1994 | The isolation and in-vivo potent antitumor-activity of clerodane diterpenoid from the oleoresin of the brazilian medicinal plant, copaifera-langsdorfii desfon | *Copaifera langsdorffii* | Anti-tumor | In vitro and in vivo | Bioorganic & medicinal chemistry letters | 66 | 2,35 | 10.1016/S0960-894X(01)80834-9 |
| DESMARCHELIER, C; COUSSIO, J; CICCIA, G | 1997 | Extracts of bolivian plants, copaifera reticulata and heisteria pallida inhibit in vitro free radical-mediated dna damage | *Copaifera reticulata* | Anti DNA damage | In vitro | Phytotherapy research | 7 | 0,28 | 10.1002/(SICI)1099-1573(199709)11:6<460::AID-PTR125>3.3.CO;2-O |
| PAIVA, LAF; RAO, VSN; GRAMOSA, NV; SILVEIRA, ER | 1998 | Gastroprotective effect of copaifera langsdorffii oleo-resin on experimental gastric ulcer models in rats | *Copaifera langsdorffii* | Gastroprotective | In vivo | Journal of ethnopharmacology | 74 | 3,08 | 10.1016/S0378-8741(98)00058-0 |
| BANDEIRA, MF; OLIVERIA, MRB; PIZZOLITTO, AC; BENATTIN, C | 2000 | Antibacterial activity of the copaiba oil associated to the ca(oh)(2) and to the zinc oxide | *Copaifera spp* | Antibacterial | In vitro | Journal of dental research | 1 | 0,045 | Not informed |
| SILVA, MAC; MELO, DFD; DE OLIVEIRA, SAM; CRUZ, AD; DA CONCEICAO, EC; DE PAULA, JR; LINO, RD; DA CUNHA, LC | 2000 | Acute and a 28-repeated dose toxicity study of commercial oleoresin from copaifera sp. In rodents | *Copaifera spp* | Tocicity | In vivo | Advances in traditional medicine | 0 | 0 | 10.1007/s13596-021-00601-7 |
| VEIGA, VF; ZUNINO, L; CALIXTO, JB; PATITUCCI, ML; PINTO, AC | 2001 | Phytochemical and antioedematogenic studies of commercial copaiba oils available in brazil | *Copaifera spp* | Antioedematogenic | In vivo | Phytotherapy research | 84 | 4 | 10.1002/ptr.976 |
| MACIEL, MAM; PINTO, AC; VEIGA, VF; GRYNBERG, NF; ECHEVARRIA, A | 2002 | Medicinal plants: the need for multidisciplinary scientific studies. | *Copaifera spp* | Anti-inflammatory, antiestrogenic, antioedema | Review paper | Quimica nova | 171 | 8,55 | 10.1590/S0100-40422002000300016 |
| TINCUSI, BM; JIMENEZ, IA; BAZZOCCHI, IL; MOUJIR, LM; MAMANI, ZA; BARROSO, JP; RAVELO, AG; HERNANDEZ, BV | 2002 | Antimicrobial terpenoids from the oleoresin of the peruvian medicinal plant copaifera paupera | *Copaifera paupera* | Antimicrobial | In vitro | Planta medica | 64 | 3,2 | 10.1055/s-2002-34399 |
| CASTRO-E-SILVA, O; RAMALHO, F; RAMALHO, L; ZUCOLOTO, S; SOUZA, ME; BRITO, M; REIS, J; BASTOS, A | 2002 | Anti-proliferative activity of oleoresin from brazilian copaifera on liver regeneration in rats | *Copaifera duckei Dwyer* | Anti-proliferative | In vivo | Journal of hepatology | 1 | 0,05 | 10.1016/S0168-8278(02)80779-6 |
| LIMA, SRM; VEIGA, VF; CHRISTO, HB; PINTO, AC; FERNANDES, PD | 2003 | In vivo and in vitro studies on the anticancer activity of copaifera multijuga hayne and its fractions | *Copaifera multijuga Hayne* | Anticancer | In vitro and in vivo | Phytotherapy research | 88 | 4,631578947 | 10.1002/ptr.1295 |
| CUNHA, KMD; PAIVA, LAF; SANTOS, FA; GRAMOSA, NV; SILVEIRA, ER; RAO, VSN | 2003 | Smooth muscle relaxant effect of kaurenoic acid, a diterpene from copaifera langsdorffii on rat uterus in vitro | *Copaifera langsdorffii* | Muscle relaxant | In vitro and in vivo | Phytotherapy research | 38 | 2 | 10.1002/ptr.1133 |
| PAIVA, LAF; GURGEL, LA; CAMPOS, AR; SILVEIRA, ER; RAO, VSN | 2004 | Attenuation of ischemia/reperfusion-induced intestinal injury by oleo-resin from copaifera langsdorffii in rats | *Copaifera langsdorffii* | Attenuation of intestinal injury | In vivo | Life sciences | 42 | 2,333333333 | 10.1016/j.lfs.2004.05.011 |
| CASTRO-E-SILVA, O; ZUCOLOTO, S; RAMALHO, FS; RAMALHO, LNZ; REIS, JMC; BASTOS, AAC; BRITO, MVH | 2004 | Antiproliferative activity of copaifera duckei oleoresin on liver regeneration in rats | *Copaifera duckei* | Antiproliferative | In vivo | Phytotherapy research | 8 | 0,444444444 | 10.1002/ptr.1351 |
| DE MENDONCA, FAC; DA SILVA, KFS; DOS SANTOS, KK; JUNIOR, KALR; SANT'ANA, AEG | 2005 | Activities of some brazilian plants against larvae of the mosquito aedes aegypti | *Copaifera langsdorffii* | Larvicidal | In vitro | Fitoterapia | 83 | 4,882352941 | 10.1016/j.fitote.2005.06.013 |
| CARVALHO, JCT; CASCON, V; POSSEBON, LS; MORIMOTO, MSS; CARDOSO, LGV; KAPLAN, MAC; GILBERT, B | 2005 | Topical antiinflammatory and analgesic activities of copaifera duckei dwyer | *Copaifera duckei Dwyer* | Antiinflammatory and analgesic | In vivo | Phytotherapy research | 44 | 2,588235294 | 10.1002/ptr.1762 |
| MAISTRO, EL; CARVALHO, JCT; CASCON, V; KAPLAN, MAC | 2005 | In vivo evaluation of the mutagenic potential and phytochemical characterization of oleoresin from copaifera duckei dwyer | *Copaifera duckei Dwyer* | Anti-mutagenic | In vivo | Genetics and molecular biology | 9 | 0,529411765 | 10.1590/S1415-47572005000500028 |
| DE LIMA, MRF; LUNA, JD; DOS SANTOS, AF; DE ANDRADE, MCC; SANT'ANA, AEG; GENET, JP; MARQUEZ, B; NEUVILLE, L; MOREAU, N | 2006 | Anti-bacterial activity of some brazilian medicinal plants | *Copaifera spp* | Antibacterial | In vitro | Journal of ethnopharmacology | 75 | 4,6875 | 10.1016/j.jep.2005.10.026 |
| VEIGA, VF; ZUNINO, L; PATITUCCI, ML; PINTO, AC; CALIXTO, JB | 2006 | The inhibition of paw oedema formation caused by the oil of copaifera multijuga hayne and its fractions | *Copaifera multijuga Hayne* | Anti-oedema | In vivo | Journal of pharmacy and pharmacology | 30 | 1,875 | 10.1111/j.2042-7158.2006.tb01659.x |
| VEIGA, VF; ROSAS, EC; CARVALHO, MV; HENRIQUES, MGMO; PINTO, AC | 2007 | Chemical composition and anti-inflammatory activity of copaiba oils from copaifera cearensis huber ex ducke, copaifera reticulata ducke and copaifera multijuga hayne - a comparative study | *Copaifera cearensis Huber ex Ducke, Copaifera reticulata Ducke and Copaifera multijuga Hayne* | Anti-inflammatory | In vivo | Journal of ethnopharmacology | 165 | 11 | 10.1016/j.jep.2007.03.005 |
| GOMES, NM; REZENDE, CM; FONTES, SP; MATHEUS, ME; FERNANDES, PD | 2007 | Antinociceptive activity of amazonian copaiba oils | *Copaifera multijuga Hayne, Copaifera reticulata Ducke* | Antinociceptive | In vitro | Journal of ethnopharmacology | 107 | 7,133333333 | 10.1016/j.jep.2006.08.018 |
| FERNANDES, FD; FREITAS, EDP | 2007 | Acaricidal activity of an oleoresinous extract from copaifera reticulata (leguminosae : caesalpinioideae) against larvae of the southern cattle tick, rhipicephalus (boophilus) microplus (acari : ixodidae) | *Copaifera reticulata* | Acaricidal | In vitro | Veterinary parasitology | 80 | 5,333333333 | 10.1016/j.vetpar.2007.02.035 |
| DA SILVA, HHG; GERIS, R; RODRIGUES, E; ROCHA, C; DA SILVA, IG | 2007 | Larvicidal activity of oil-resin fractions from the brazilian medicinal plant copaifera reticulata ducke (leguminosae-caesalpinoideae) against aedes aegypti (diptera, culicidae) | *Copaifera reticulata Ducke* | Insecticidal | In vitro | Revista da sociedade brasileira de medicina tropical | 20 | 1,333333333 | 10.1590/S0037-86822007000300002 |
| DOS SANTOS, O; UEDA-NAKAMURA, T; FILHO, PDB | 2007 | Antimicrobial activity of copaiba oils obtained from different species of copaifera in brazil | *Copaifera martii, Copaifera officinalis, and Copaifera reticulata* | Antimicrobial | In vitro | Planta medica | 1 | 0,066666667 | Not informed |
| DOS SANTOS, AO; UEDA-NAKAMURA, T; DIAS, BP; VEIGA, VF; PINTO, AC; NAKAMURA, CV | 2008 | Antimicrobial activity of brazilian copaiba oils obtained from different species of the copaifera genus | *C. Multijuga Hayne, C. Officinalis Jacq, C. Reticulata Ducke, C. Lucens, C. Langsdorffii Desf, C. Paupera Dwyer, C. Martii, C. Cearensis Huber* | Antibacterial and antifungal | In vitro | Memorias do instituto oswaldo cruz | 109 | 7,785714286 | 10.1590/S0074-02762008005000015 |
| SANTOS, AO; UEDA-NAKAMURA, T; DIAS, BP; VEIGA, VF; PINTO, AC; NAKAMURA, CV | 2008 | Effect of brazilian copaiba oils on leishmania amazonensis | *Copaifera multijuga Hayne, Copaifera officinalis Jacq, Copaifera reticulata Ducke,* | Antiparasitic | In vitro | Journal of ethnopharmacology | 99 | 7,071428571 | 10.1016/j.jep.2008.08.007 |
| GOMES, ND; REZENDE, CD; FONTES, SP; HOVELL, AMC; LANDGRAF, RG; MATHEUS, ME; PINTO, AD; FERNANDES, PD | 2008 | Antineoplasic activity of copaifera multijuga oil and fractions against ascitic and solid ehrlich tumor | *Copaifera multijuga* | Antineoplasic | In vitro | Journal of ethnopharmacology | 42 | 3 | 10.1016/j.jep.2008.06.033 |
| CORREIA, AF; SEGOVIA, JFO; GONCALVES, MCA; DE OLIVEIRA, VL; SILVEIRA, D; CARVALHO, JCT; KANZAKI, LIB | 2008 | Amazonian plant crude extract screening for activity against multidrug-resistant bacteria | *Copaifera spp* | Antibacterial | Review paper | European review for medical and pharmacological sciences | 23 | 1,642857143 | Not informed |
| VASCONCELOS, KRF; DA VEIGA, VF; ROCHA, WC; BANDEIRA, MFCL | 2008 | In vitro assessment of antibacterial activity of a dental cement constituted of a copaifera multijuga hayne oil-resin | *Copaifera multijuga Hayne* | Antibacterial | In vitro | Revista brasileira de farmacognosia-brazilian journal of pharmacognosy | 22 | 1,571428571 | 10.1590/S0102-695X2008000500017 |
| PEREIRA, FJ; MARTINS, FT; CORREA, RS; MOREIRA, MEC; COSTA, AMDD; DOS SANTOS, MH; POLO, M; BARBOSA, LCA | 2008 | Isolation, chemical composition and anti-inflammatory activity of copaifera langsdorffii desf. Fruit peels essential oil according to successive hydrodistillations | *Copaifera langsdorffii* | Anti-inflammatory | In vivo | Latin american journal of pharmacy | 5 | 0,357142857 | Not informed |
| MENDONCA, DE; ONOFRE, SB | 2009 | Antimicrobial activity of the oil-resin produced by copaiba copaifera multijuga hayne (leguminosae). | *Copaifera multijuga Hayne* | Antimicrobial | In vitro | Revista brasileira de farmacognosia-brazilian journal of pharmacognosy | 31 | 2,384615385 | 10.1590/S0102-695X2009000400012 |
| SILVA, JJD; GUIMARAES, SB; DA SILVEIRA, ER; DE VASCONCELOS, PRL; LIMA, GG; TORRES, SM; DE VASCONCELOS, RC | 2009 | Effects of copaifera langsdorffii desf. On ischemia-reperfusion of randomized skin flaps in rats | *Copaifera langsdorffii Desf* | Anti-inflammatory and antioxidant | In vivo | Aesthetic plastic surgery | 26 | 2 | 10.1007/s00266-008-9263-2 |
| DOS SANTOS, AB; SILVA, DHS; BOLZANI, VD; SANTOS, LA; SCHMIDT, TM; BAFFA, O | 2009 | Antioxidant properties of plant extracts: an epr and dft comparative study of the reaction with dpph, tempol and spin trap dmpo | *Copaifera langsdorffii* | Antioxidant | In vitro | Journal of the brazilian chemical society | 25 | 1,923076923 | 10.1590/S0103-50532009000800015 |
| DA SILVA, AC; SALES, NDP; DE ARAUJO, AV; CALDEIRA, CF | 2009 | In vitro effect of plant compounds on the fungus colletotrichum gloeosporioides penz. Isolated from passion fruit | *Copaifera langsdorffi* | Antifungal | In vitro | Ciencia e agrotecnologia | 21 | 1,615384615 | 10.1590/S1413-70542009000700026 |
| SACHETTI, CG; FASCINELI, ML; SAMPAIO, JA; LAMEIRA, OA; CALDAS, ED | 2009 | Assessment of the neurotoxic potential and acute toxicity of copaiba. | *Copaifera reticulata Ducke* | Neurotoxicity | In vivo | Revista brasileira de farmacognosia-brazilian journal of pharmacognosy | 14 | 1,076923077 | 10.1590/S0102-695X2009000600025 |
| ESTEVAO, LRD; DE MEDEIROS, JP; SCOGNAMILLO-SZABO, MVR; BARATELLA-EVENCIO, L; GUIMARAES, EC; DA CAMARA, CAG; EVENCIO-NETO, J | 2009 | Neoangiogenesis of skin flaps in rats treated with copaiba oil | *Copaifera langsdorffii* | Healing | In vivo | Pesquisa agropecuaria brasileira | 12 | 0,923076923 | Not informed |
| CURIO, M; JACONE, H; PERRUT, J; PINTO, AC; VEIGA, VF; SILVA, RCB | 2009 | Acute effect of copaifera reticulata ducke copaiba oil in rats tested in the elevated plus-maze: an ethological analysis | *Copaifera reticulata Ducke* | Anxiolytic | In vivo | Journal of pharmacy and pharmacology | 8 | 0,615384615 | 10.1211/jpp/61.08.0015 |
| GOMES, ND; DE REZENDE, CM; FONTES, SP; MATHEUS, ME; PINTO, AD; FERNANDES, PD | 2010 | Characterization of the antinociceptive and anti-inflammatory activities of fractions obtained from copaifera multijuga hayne | *Copaifera multijuga Hayne* | Antinociceptive and anti-inflammatory | In vivo | Journal of ethnopharmacology | 49 | 4,083333333 | 10.1016/j.jep.2010.01.005 |
| PIERI, FA; MUSSI, MC; FIORINI, JE; SCHNEEDORF, JM | 2010 | Clinical and microbiological effects of copaiba oil (copaifera officinalis) on dental plaque forming bacteria in dogs | *Copaifera officinalis* | Antimicrobial | In vivo | Arquivo brasileiro de medicina veterinaria e zootecnia | 15 | 1,25 | 10.1590/S0102-09352010000300012 |
| SOUZA, APS; GURGEL, ESC; QUEIROZ, MSM; SANTOS, JUM | 2010 | Allelophatic activity of crude extracts from three species of copaifera (leguminosae-caesalpinioidedae) | *Copaifera duckei, C. Martii and C. Reticulata* | Allelophatic | In vitro | Planta daninha | 14 | 1,166666667 | 10.1590/S0100-83582010000400006 |
| COMELLI, E; SKINOVSKI, J; SIGWALT, MF; BRANCO, AB; LUZ, SR; BAULE, CD | 2010 | Rupture point analysis of intestinal anastomotic healing in rats under the action of pure copaiba (copaifera iangsdorfii) oil | *Copaifera langsdorffii* | Strength of digestive tract scar | In vivo | Acta cirurgica brasileira | 13 | 1,083333333 | 10.1590/S0102-86502010000400012 |
| PIERI, FA; JOSE, RM; GALVAO, NN; NERO, LA; MOREIRA, MAS | 2010 | Antimicrobial activity of autoclaved and non autoclaved copaiba oil on listeria monocytogenes | *Copaifera langsdorffii* | Antimicrobial | In vitro | Ciencia rural | 11 | 0,916666667 | 10.1590/S0103-84782010000800020 |
| DE OLIVEIRA, RVM; OHARA, MT; VILA, MMDC; GONCALVES, MM | 2010 | In vitro evaluation of copaiba oil as a kojic acid skin enhancer | *Copaifera spp* | Skin enhancer | In vitro | Brazilian journal of pharmaceutical sciences | 4 | 0,333333333 | 10.1590/S1984-82502010000200024 |
| SOUZA, AB; MARTINS, CHG; SOUZA, MGM; FURTADO, NAJC; HELENO, VCG; DE SOUSA, JPB; ROCHA, EMP; BASTOS, JK; CUNHA, WR; VENEZIANI, RCS; AMBROSIO, SR | 2011 | Antimicrobial activity of terpenoids from copaifera langsdorffii desf. Against cariogenic bacteria | *Copaifera langsdorffii* | Antibacterial | In vitro | Phytotherapy research | 82 | 7,454545455 | 10.1002/ptr.3244 |
| SOUZA, AB; DE SOUZA, MGM; MOREIRA, MA; MOREIRA, MR; FURTADO, NAJC; MARTINS, CHG; BASTOS, JK; DOS SANTOS, RA; HELENO, VCG; AMBROSIO, SR; VENEZIANI, RCS | 2011 | Antimicrobial evaluation of diterpenes from copaifera langsdorffii oleoresin against periodontal anaerobic bacteria | *Copaifera langsdorffii* | Antibacterial | In vitro | Molecules | 72 | 6,545454545 | 10.3390/molecules16119611 |
| POHLIT, AM; REZENDE, AR; BALDIN, ELL; LOPES, NP; NETO, VFD | 2011 | Plant extracts, isolated phytochemicals, and plant-derived agents which are lethal to arthropod vectors of human tropical diseases - a review | *Copaifera spp* | Anti Arthropods | Review paper | Planta medica | 66 | 6 | 10.1055/s-0030-1270949 |
| DOS SANTOS, AO; COSTA, MA; UEDA-NAKAMURA, T; DIAS, BP; DA VEIGA, VF; LIMA, MMD; NAKAMURA, CV | 2011 | Leishmania amazonensis: effects of oral treatment with copaiba oil in mice | *Copaifera martii* | Antileishmanial | In vivo | Experimental parasitology | 41 | 3,727272727 | 10.1016/j.exppara.2011.06.016 |
| KOBAYASHI, C; FONTANIVE, TO; ENZWEILER, BG; DE BONA, LR; MASSONI, T; APEL, MA; HENRIQUES, AT; RICHTER, MF; ARDENGHI, P; SUYENAGA, ES | 2011 | Pharmacological evaluation of copaifera multijuga oil in rats | *Copaifera multijuga* | Anti-inflammatory | In vivo | Pharmaceutical biology | 22 | 2 | 10.3109/13880209.2010.515595 |
| VALOTTO, CFB; DA SILVA, HHG; CAVASIN, G; GERIS, R; RODRIGUES, E; DA SILVA, IG | 2011 | Ultrastructural alterations in larvae of aedes aegypti subject to labdane diterpene isolated from copaifera reticulata (leguminosae) and a fraction enriched with tannins of magonia pubescens (sapindaceae) | *Copaifera reticulata* | Larvicidal | In vitro | Revista da sociedade brasileira de medicina tropical | 19 | 1,727272727 | 10.1590/S0037-86822011005000010 |
| SACHETTI, CG; DE CARVALHO, RR; PAUMGARTTEN, FJR; LAMEIRA, OA; CALDAS, ED | 2011 | Developmental toxicity of copaiba tree (copaifera reticulata ducke, fabaceae) oleoresin in rat | *Copaifera reticulata Ducke* | Toxicity | In vivo | Food and chemical toxicology | 19 | 1,727272727 | 10.1016/j.fct.2011.01.015 |
| BARBOSA, FS; LEITE, GLD; ALVES, SM; NASCIMENTO, AF; D'AVILA, VD; DA COSTA, CA | 2011 | Insecticide effects of ruta graveolens, copaifera langsdorffii and chenopodium ambrosioides against pests and natural enemies in commercial tomato plantation | *Copaifera langsdorffii* | Insecticidal | Human reports | Acta scientiarum-agronomy | 18 | 1,636363636 | 10.4025/actasciagron.v33i1.5900 |
| NETO, JN; LINDOSO, MJD; COELHO, LF; CARVALHO, RAF; RODRIGUES, TGPD; DE ARAUJO, AGP; GIRAO, MJBC; SCHOR, E | 2011 | Changes in the volume and histology of endometriosis foci in rats treated with copaiba oil (copaifera langsdorffii) | *Copaifera langsdorffii* | Anti-endometriosis | In vivo | Acta cirurgica brasileira | 15 | 1,363636364 | 10.1590/S0102-86502011000800005 |
| PIERI, FA; SOUZA, CF; COSTA, JCM; BARRERO, MAO; ESPESCHIT, IF; SILVA, VO; MOREIRA, MAS | 2011 | Inhibition of escherichia coli from mastitic milk by copaiba oil | *Copaifera langsdorffii and Copaifera officinalis* | Antibacterial | In vitro | Semina-ciencias agrarias | 7 | 0,636363636 | 10.5433/1679-0359.2011v32Splp1929 |
| LEANDRO, LM; VARGAS, FD; BARBOSA, PCS; NEVES, JKO; DA SILVA, JA; DA VEIGA, VF | 2012 | Chemistry and biological activities of terpenoids from copaiba (copaifera spp.) Oleoresins | *Copaifera spp* | Antimicrobial, Anti-inflammatory, Gastroprotective, Healing, Antioxidant, antitumor | Review paper | Molecules | 150 | 15 | 10.3390/molecules17043866 |
| SANTOS, RCV; ALVES, CFD; SCHNEIDER, T; LOPES, LQS; AURICH, C; GIONGO, JL; BRANDELLI, A; VAUCHER, RD | 2012 | Antimicrobial activity of amazonian oils against paenibacillus species | *Copaifera officinalis* | Antimicrobial | In vitro | Journal of invertebrate pathology | 42 | 4,2 | 10.1016/j.jip.2011.12.002 |
| GUIMARAES-SANTOS, A; SANTOS, DS; SANTOS, IR; LIMA, RR; PEREIRA, A; DE MOURA, LS; CARVALHO, RN; LAMEIRA, O; GOMES-LEAL, A | 2012 | Copaiba oil-resin treatment is neuroprotective and reduces neutrophil recruitment and microglia activation after motor cortex excitotoxic injury | *Copaifera spp* | Neuroprotective | In vivo | Evidence-based complementary and alternative medicine | 42 | 4,2 | 10.1155/2012/918174 |
| PROPHIRO, JS; DA SILVA, MAN; KANIS, LA; DA SILVA, BM; DUQUE-LUNA, JE; DA SILVA, OS | 2012 | Evaluation of time toxicity, residual effect, and growth-inhibiting property of carapa guianensis and copaifera sp in aedes aegypti | *Copaifera spp* | Larvicidal | In vitro | Parasitology research | 34 | 3,4 | 10.1007/s00436-011-2547-5 |
| PROPHIRO, JS; DA SILVA, MAN; KANIS, LA; DA ROCHA, LCBP; DUQUE-LUNA, JE; DA SILVA, OS | 2012 | First report on susceptibility of wild aedes aegypti (diptera: culicidae) using carapa guianensis (meliaceae) and copaifera sp (leguminosae) | *Copaifera spp* | Antilarval | In vitro | Parasitology research | 33 | 3,3 | 10.1007/s00436-011-2545-7 |
| BRANCALION, APS; OLIVEIRA, RB; SOUSA, JPB; GROPPO, M; BERRETTA, AA; BARROS, ME; BOIM, MA; BASTOS, JK | 2012 | Effect of hydroalcoholic extract from copaifera langsdorffii leaves on urolithiasis induced in rats | *Copaifera langsdorffii* | Anti-urolithiasis | In vivo | Urological research | 33 | 3,3 | 10.1007/s00240-011-0453-z |
| ALMEIDA, MR; DARIN, JDC; HERNANDES, LC; RAMOS, MFD; ANTUNES, LMG; DE FREITAS, O | 2012 | Genotoxicity assessment of copaiba oil and its fractions in swiss mice | *Copaifera spp* | Genotoxicity | In vivo | Genetics and molecular biology | 27 | 2,7 | 10.1590/S1415-47572012005000052 |
| KANIS, LA; PROPHIRO, JS; VIEIRA, ED; DO NASCIMENTO, MP; ZEPON, KM; KULKAMP-GUERREIRO, IC; DA SILVA, OS | 2012 | Larvicidal activity of copaifera sp (leguminosae) oleoresin microcapsules against aedes aegypti (diptera: culicidae) larvae | *Copaifera spp* | Larvicidal | In vitro | Parasitology research | 21 | 2,1 | 10.1007/s00436-011-2610-2 |
| DOS SANTOS, AO; UEDA-NAKAMURA, T; DIAS, BP; DA VEIGA, VF; NAKAMURA, CV | 2012 | Copaiba oil: an alternative to development of new drugs against leishmaniasis | *Copaifera reticulata Ducke* | Antileishmanial | In vitro | Evidence-based complementary and alternative medicine | 20 | 2 | 10.1155/2012/898419 |
| RONDON, FCM; BEVILAQUA, CML; ACCIOLY, MP; DE MORAIS, SM; DE ANDRADE, HF; DE CARVALHO, CA; LIMA, JC; MAGALHAES, HCR | 2012 | In vitro efficacy of coriandrum sativum, lippia sidoides and copaifera reticulata against leishmania chagasi | *Copaifera reticulata* | Antilehmania | In vitro | Revista brasileira de parasitologia veterinaria | 18 | 1,8 | 10.1590/S1984-29612012000300002 |
| DA SILVA, AG; PUZIOL, PD; LEITAO, RN; GOMES, TR; SCHERER, R; MARTINS, MLL; CAVALCANTI, ASS; CAVALCANTI, LC | 2012 | Application of the essential oil from copaiba (copaifera langsdorffii desf.) For acne vulgaris: a double-blind, placebo controlled clinical trial | *Copaifera langsdorffii* | Antiacne | Human reports | Alternative medicine review | 14 | 1,4 | Not informed |
| PIERI, FA; SILVA, VO; SOUZA, CF; COSTA, JCM; SANTOS, LF; MOREIRA, MAS | 2012 | Antimicrobial profile screening of two oils of copaifera genus | *C. Langsdorffii e Copaifera officinalis* | Antimicrobial | In vitro | Arquivo brasileiro de medicina veterinaria e zootecnia | 12 | 1,2 | 10.1590/S0102-09352012000100037 |
| GELMINI, F; BERETTA, G; ANSELMI, C; CENTINI, M; MAGNI, P; RUSCICA, M; CAVALCHINI, A; FACINO, RM | 2013 | Gc-ms profiling of the phytochemical constituents of the oleoresin from copaifera langsdorffii desf. And a preliminary in vivo evaluation of its antipsoriatic effect | *Copaifera langsdorffii* | Antipsoriatic | In vitro | International journal of pharmaceutics | 59 | 6,555555556 | 10.1016/j.ijpharm.2012.08.021 |
| ALVES, JM; MUNARI, CC; NETO, MDBM; FURTADO, RA; SENEDESE, JM; BASTOS, JK; TAVARES, DC | 2013 | In vivo protective effect of copaifera langsdorffii hydroalcoholic extract on micronuclei induction by doxorubicin | *Copaifera lansdorffii* | Genotoxicity | In vivo | Journal of applied toxicology | 27 | 3 | 10.1002/jat.2777 |
| SENEDESE, JM; ALVES, JM; LIMA, IMD; DE ANDRADE, EAP; FURTADO, RA; BASTOS, JK; TAVARES, DC | 2013 | Chemopreventive effect of copaifera langsdorffii leaves hydroalcoholic extract on 1,2-dimethylhydrazine-induced dna damage and preneoplastic lesions in rat colon | *Copaifera langsdorffii* | Chemopreventive | In vivo | Bmc complementary and alternative medicine | 25 | 2,777777778 | 10.1186/1472-6882-13-3 |
| ZIMMERMAM-FRANCO, DC; BOLUTARI, EB; POLONINI, HC; DO CARMO, AMR; CHAVES, MDGAM; RAPOSO, NRB | 2013 | Antifungal activity of copaifera langsdorffii desf oleoresin against dermatophytes | *Copaifera langsdorffii* | Antifungal | In vitro | Molecules | 23 | 2,555555556 | 10.3390/molecules181012561 |
| ESTEVAO, LRM; DE MEDEIROS, JP; BARATELLA-EVENCIO, L; SIMOES, RS; MENDONCA, FD; EVENCIO-NETO, J | 2013 | Effects of the topical administration of copaiba oil ointment (copaifera langsdorffii) in skin flaps viability of rats | *Copaifera langsdorffii* | Dermatogenic | In vivo | Acta cirurgica brasileira | 22 | 2,444444444 | 10.1590/S0102-86502013001200009 |
| TRINDADE, FTT; STABELI, RG; PEREIRA, AA; FACUNDO, VA; SILVA, ADE | 2013 | Copaifera multijuga ethanolic extracts, oil-resin, and its derivatives display larvicidal activity against anopheles darlingi and aedes aegypti (diptera: culicidae) | *Copaifera multijuga* | Antilarval | In vitro | Revista brasileira de farmacognosia-brazilian journal of pharmacognosy | 21 | 2,333333333 | 10.1590/S0102-695X2013005000038 |
| DOS SANTOS, ECG; DONNICI, CL; CAMARGOS, ERD; DE REZENDE, AA; ANDRADE, EHD; SOARES, LAL; FARIAS, LD; DE CARVALHO, MAR; ALMEIDA, MDG | 2013 | Effects of copaifera duckei dwyer oleoresin on the cell wall and cell division of bacillus cereus | *Copaifera duckei Dwyer* | Antibacterial | In vitro | Journal of medical microbiology | 16 | 1,777777778 | 10.1099/jmm.0.060046-0 |
| ZIECH, RE; FARIAS, LD; BALZAN, C; ZIECH, MF; HEINZMANN, BM; LAMEIRA, OA; DE VARGAS, AC | 2013 | Antimicrobial activity of copaiba oil (copaifera reticulata) against coagulase positive staphylococcus of canine otitis | *Copaifera reticulata* | Antimicrobial | In vivo | Pesquisa veterinaria brasileira | 12 | 1,333333333 | 10.1590/S0100-736X2013000700011 |
| IZUMI, E; UEDA-NAKAMURA, T; VEIGA, VF; NAKAMURA, CV | 2013 | Toxicity of oleoresins from the genus copaifera in trypanosoma cruzi: a comparative study | *Copaifera reticulata, C. Martii, C. Langsdorffii, C. Paupera, C. Multijuga, , C. Officinalis, C. Lucens* | Anti-Trypanosoma cruzi | In vitro | Planta medica | 11 | 1,222222222 | 10.1055/s-0032-1328712 |
| YASOJIMA, EY; TEIXEIRA, RKC; HOUAT, AD; COSTA, FLD; SILVEIRA, EL; BRITO, MVH; LOPES, GD | 2013 | Effect of copaiba oil on correction of abdominal wall defect treated with the use of polypropylene/polyglecaprone mesh | *Copaifera spp* | Healing | In vivo | Acta cirurgica brasileira | 11 | 1,222222222 | 10.1590/S0102-86502013000200008 |
| DE OLIVEIRA, RB; COELHO, EB; RODRIGUES, MR; COSTA-MACHADO, ARD; DE SOUSA, JPB; BERRETTA, AA; BASTOS, JK | 2013 | Effect of the copaifera langsdorffii desf. Leaf extract on the ethylene glycol-induced nephrolithiasis in rats | *Copaifera langsdorffii* | Prevent kidney stone formation | In vivo | Evidence-based complementary and alternative medicine | 10 | 1,111111111 | 10.1155/2013/131372 |
| DORNELES, FDS; DA SILVA, AS; OLIVEIRA, CB; ZIMMERMANN, CEP; ROSA, LD; TONIN, AA; DE OLIVEIRA, ECP; SANTURIO, JM; MONTEIRO, SG | 2013 | Susceptibility of trypanosoma evansi in the copaiba oil: in vitro test and in mice experimentally infected with the parasite | *Copaifera reticulata and Copaifera ducke* | Anti-Trypanosoma | In vivo | Acta scientiae veterinariae | 5 | 0,555555556 | Not informed |
| TEIXEIRA, RKC; YAMAKI, VN; YASOJIMA, EY; BRITO, MVH | 2013 | Effect of copaiba oil in hepatic damage induced by acetaminophen in rats | *Copaiffera officinalis* | Reducing liver damage caused by paracetamol | In vivo | Acta cirurgica brasileira | 4 | 0,444444444 | 10.1590/S0102-86502013000700008 |
| BOTELHO, NM; CORREA, SC; LOBATO, RC; TEIXEIRA, RKC; QUARESMA, JAS | 2013 | Immunohistochemistry of the uterine cervix of rats bearing the walker 256 tumor treated with copaiba balsam | *Copaifera officinalis* | Anti-tumor | In vivo | Acta cirurgica brasileira | 2 | 0,222222222 | 10.1590/S0102-86502013000300005 |
| ANDRADE, BFMT; BARBOSA, LN; PROBST, ID; FERNANDES, A | 2014 | Antimicrobial activity of essential oils | *Copaifera officinalis* | Antimicrobial | In vitro | Journal of essential oil research | 91 | 11,375 | 10.1080/10412905.2013.860409 |
| DIAS, DS; FONTES, LBA; CROTTI, AEM; AARESTRUP, BJV; AARESTRUP, FM; DA SILVA, AA; CORREA, JOA | 2014 | Copaiba oil suppresses inflammatory cytokines in splenocytes of c57bl/6 mice induced with experimental autoimmune encephalomyelitis (eae) | *Copaifera officinalis* | Anti-inflammatory | In vivo | Molecules | 25 | 3,125 | 10.3390/molecules190812814 |
| RODRIGUES, EDR; FERREIRA, AM; VILHENA, JCE; ALMEIDA, FB; CRUZ, RAS; FLORENTINO, AC; SOUTO, RNP; CARVALHO, JCT; FERNANDES, CP | 2014 | Development of a larvicidal nanoemulsion with copaiba (copaifera duckei) oleoresin | *Copaifera reticulata duckey* | Larvicidal | In vitro | Revista brasileira de farmacognosia-brazilian journal of pharmacognosy | 25 | 3,125 | 10.1016/j.bjp.2014.10.013 |
| DESTRYANA, RA; YOUNG, DG; WOOLLEY, CL; HUANG, TC; WU, HY; SHIH, WL | 2014 | Antioxidant and anti-inflammation activities of ocotea, copaiba and blue cypress essential oils in vitro and in vivo | *Copaifera reticulata* | Anti-inflammatory and antioxidant | In vitro and in vivo | Journal of the american oil chemists society | 15 | 1,875 | 10.1007/s11746-014-2504-4 |
| PIERI, FA; SILVA, VO; VARGAS, FS; VEIGA, VF; MOREIRA, MAS | 2014 | Antimicrobial activity of copaifera langsdorffii oil and evaluation of its most bioactive fraction against bacteria of dog's dental plaque | *Copaifera langsdorffii* | Antibacterial | In vitro | Pakistan veterinary journal | 10 | 1,25 | Not informed |
| GONCALVES, ES; SILVA, JR; GOMES, CL; NERY, MBL; NAVARRO, DMAF; SANTOS, GKN; SILVA-NETO, JC; COSTA-SILVA, JH; ARAUJO, AV; WANDERLEY, AG | 2014 | Effects of the oral treatment with copaifera multijuga oil on reproductive performance of male wistar rats | *Copaifera multijuga* | Reproductive performance | In vivo | Revista brasileira de farmacognosia-brazilian journal of pharmacognosy | 8 | 1 | 10.1016/j.bjp.2014.07.014 |
| TEIXEIRA, LF; BOAS, RV; OLIVEIRA, PC; DE CASTRO, HF | 2014 | Effect of natural antioxidants on the lipase activity in the course of batch and continuous glycerolysis of babassu oil | *Copaifera spp* | Lipase activity | In vitro | Bioprocess and biosystems engineering | 5 | 0,625 | 10.1007/s00449-014-1144-2 |
| BALDISSERA, MD; DA SILVA, AS; OLIVEIRA, CB; VAUCHER, RA; SANTOS, RCV; GIONGO, JL; CAPELETO, DM; TONIN, AA; MONTEIRO, SG | 2014 | Using of essential oils in the treatment of mice infected with trypanosoma evansi | *Copaifera officinalis* | Anti-Trypanosoma | In vivo | Revista mvz cordoba | 3 | 0,375 | Not informed |
| MONDEGO, JM; DE MELO, PAFR; PINTO, KMS; DO NASCIMENTO, LC; ALVES, EU; BATISTA, JD | 2014 | Alternative control of microflora on pseudobombax marginatum seeds with essential copaiba oil (copaifera sp.) | *Copaifera spp* | Antifungal | In vitro | Bioscience journal | 2 | 0,25 | Not informed |
| BRITO, MVH; COSTA, FD; DE VASCONCELOS, DM; COSTA, LAV; YASOJIMA, EY; TEIXEIRA, RKC; YAMAKI, VN | 2014 | Attenuation of copaiba oil in hepatic damage in rats | *Copaifera officinalis* | Reducing liver damage caused by paracetamol | In vivo | Acta cirurgica brasileira | 2 | 0,25 | 10.1590/S0102-86502014001900002 |
| NETO, SED; FRANCISCO, WD; NETO, RDA; LUSTOSA, C; RIBEIRO, SAL | 2014 | Anthracnose postharvest control in yellow passion fruit with application of copaiba oil | *Copaifera spp* | Anthracnose | In vitro | Revista brasileira de fruticultura | 0 | 0 | 10.1590/0100-2945-146/13 |
| BONAN, RF; BONAN, PRF; BATISTA, AUD; SAMPAIO, FC; ALBUQUERQUE, AJR; MORAES, MCB; MATTOSO, LHC; GLENN, GM; MEDEIROS, ES; OLIVEIRA, JE | 2015 | In vitro antimicrobial activity of solution blow spun poly(lactic acid)/polyvinylpyrrolidone nanofibers loaded with copaiba (copaifera sp.) Oil | *Copaifera spp* | Antimicrobial | In vitro | Materials science & engineering c-materials for biological applications | 61 | 8,714285714 | 10.1016/j.msec.2014.12.021 |
| MORELLI, CL; MAHROUS, M; BELGACEM, MN; BRANCIFORTI, MC; BRETAS, RES; BRAS, J | 2015 | Natural copaiba oil as antibacterial agent for bio-based active packaging | *Copaiferamultijuga* | Antibacterial | In vitro | Industrial crops and products | 41 | 5,857142857 | 10.1016/j.indcrop.2015.03.036 |
| ALENCAR, EN; XAVIER, FH; MORAIS, ARV; DANTAS, TRF; DANTAS-SANTOS, N; VERISSIMO, LM; REHDER, VLG; CHAVES, GM; OLIVEIRA, AG; EGITO, EST | 2015 | Chemical characterization and antimicrobial activity evaluation of natural oil nanostructured emulsions | *Copaifera langsdorffii* | Antimicrobial | In vitro | Journal of nanoscience and nanotechnology | 30 | 4,285714286 | 10.1166/jnn.2015.9187 |
| ABRAO, F; COSTA, LDD; ALVES, JM; SENEDESE, JM; DE CASTRO, PT; AMBROSIO, SR; VENEZIANI, RCS; BASTOS, JK; TAVARES, DC; MARTINS, CHG | 2015 | Copaifera langsdorffii oleoresin and its isolated compounds: antibacterial effect and antiproliferative activity in cancer cell lines | *Copaifera langsdorffii* | Antibacterial and anticancer | In vitro | Bmc complementary and alternative medicine | 26 | 3,714285714 | 10.1186/s12906-015-0961-4 |
| LEMOS, M; SANTIN, JR; MIZUNO, CS; BOEING, T; DE SOUSA, JPB; NANAYAKKARA, D; BASTOS, JK; DE ANDRADE, SF | 2015 | Copaifera langsdorffii: evaluation of potential gastroprotective of extract and isolated compounds obtained from leaves | *Copaifera langsdorffi* | Gastroprotective | In vitro | Revista brasileira de farmacognosia-brazilian journal of pharmacognosy | 26 | 3,714285714 | 10.1016/j.bjp.2015.05.005 |
| SVETLICHNY, G; KULKAMP-GUERREIRO, IC; CUNHA, SL; SILVA, FEK; BUENO, K; POHLMANN, AR; FUENTEFRIA, AM; GUTERRES, SS | 2015 | Solid lipid nanoparticles containing copaiba oil and allantoin: development and role of nanoencapsulation on the antifungal activity | *Copaifera spp* | Antifungal | In vitro | Pharmazie | 25 | 3,571428571 | 10.1691/ph.2015.4116 |
| SANTIAGO, KB; CONTI, BJ; ANDRADE, BFMT; DA SILVA, JJM; ROGEZ, HLG; CREVELIN, EJ; DE MORAES, LAB; VENEZIANI, R; AMBROSIO, SR; BASTOS, JK; SFORCIN, JM | 2015 | Immunomodulatory action of copaifera spp oleoresins on cytokine production by human monocytes | *Copaifera reticulata, Copaifera duckey and Copaifera multijuga* | Immunomodulatory action | In vitro | Biomedicine & pharmacotherapy | 23 | 3,285714286 | 10.1016/j.biopha.2014.12.035 |
| BOTELHO, JRS; SANTOS, AG; ARAUJO, ME; BRAGA, MEM; GOMES-LEAL, W; CARVALHO, RN; MEIRELES, MAA; OLIVEIRA, MS | 2015 | Copaiba (copaifera sp.) Leaf extracts obtained by co2 supercritical fluid extraction: isotherms of global yield, kinetics data, antioxidant activity and neuroprotective effects | *Copaifera spp* | Antioxidant activity and neuroprotective effects | In vitro and in vivo | Journal of supercritical fluids | 20 | 2,857142857 | 10.1016/j.supflu.2014.12.006 |
| FURTADO, RA; BERNARDES, CTV; DA SILVA, MN; ZOCCAL, KF; FACCIOLI, LH; BASTOS, JK | 2015 | Antiedematogenic evaluation of copaifera langsdorffii leaves hydroethanolic extract and its major compounds | *Copaifera langsdorffii* | Antiedematogenic | In vitro and in vivo | Biomed research international | 15 | 2,142857143 | 10.1155/2015/913152 |
| VAUCHER, RD; GIONGO, JL; BOLZAN, LP; CORREA, MS; FAUSTO, VP; ALVES, CFD; LOPES, LQS; BOLIGON, AA; ATHAYDE, ML; MOREIRA, AP; BRANDELLI, A; RAFFIN, RP; SANTOS, RCV | 2015 | Antimicrobial activity of nanostructured amazonian oils against paenibacillus species and their toxicity on larvae and adult worker bees | *Copaifera-multijuga hayne* | Antimicrobial | In vitro | Journal of asia-pacific entomology | 14 | 2 | 10.1016/j.aspen.2015.01.004 |
| ADZU, B; BALOGUN, SO; PAVAN, E; ASCENCIO, SD; SOARES, IM; AGUIAR, RWS; RIBEIRO, RV; BESERRA, AMSES; DE OLIVEIRA, RG; DA SILVA, LI; DAMAZO, AS; MARTINS, DTD | 2015 | Evaluation of the safety, gastroprotective activity and mechanism of action of standardised leaves infusion extract of copaifera malmei harms | *Copaifera malmei* | Gastroprotectionand anti-ulcer | In vivo | Journal of ethnopharmacology | 13 | 1,857142857 | 10.1016/j.jep.2015.09.027 |
| VOLPATO, A; GROSSKOPF, RK; SANTOS, RC; VAUCHER, RA; RAFFIN, RP; BOLIGON, AA; ATHAYDE, ML; STEFANI, LM; DA SILVA, AS | 2015 | Influence of rosemary, andiroba and copaiba essential oils on different stages of the biological cycle of the tick rhipicephalus microplus in vitro | *Copaifera officinalis* | Acaricidal | In vitro | Journal of essential oil research | 5 | 0,714285714 | 10.1080/10412905.2015.1010045 |
| DIAS, FGG; CASEMIRO, LA; MARTINS, CHG; DIAS, LGGG; PEREIRA, LD; NISHIMURA, LT; DE SOUZA, FF; HONSHO, CD | 2015 | Endodontics pastes formulated with copaiba oil: action on oral microbiota and dentin bridge formation in dogs | *Copaifera langsdorffii Desfon* | Antimicrobial | In vivo | Ciencia rural | 3 | 0,428571429 | 10.1590/0103-8478cr20141284 |
| LOPES, LN; SANTOS, FAF; OLIVEIRA, LCM; PERCARIO, S; DE BARROS, CAV; BRITO, MVH | 2015 | Copaiba oil effect on induced fecal peritonitis in rats | *Copaifera officinalis* | Increasing survival of rats subjected to severe sepsis | In vivo | Acta cirurgica brasileira | 3 | 0,428571429 | 10.1590/S0102-865020150080000008 |
| SILVA, PF; BRITO, MVH; PONTES, FSC; RAMOS, SR; MENDES, LC; OLIVEIRA, LCM | 2015 | Copaiba oil effect on experimental jaw defect in wistar rats | *Copaifera spp* | Anti-inflammatory and healing | In vivo | Acta cirurgica brasileira | 1 | 0,142857143 | 10.1590/S0102-86502015002000006 |
| BARDAJI, DKR; DA SILVA, JJM; BIANCHI, TC; EUGENIO, DD; DE OLIVEIRA, PF; LEANDRO, LF; ROGEZ, HLG; VENEZIANNI, RCS; AMBROSIO, SR; TAVARES, DC; BASTOS, JK; MARTINS, CHG | 2016 | Copaifera reticulata oleoresin: chemical characterization and antibacterial properties against oral pathogens | *Copaifera reticulata* | Antibacterial | In vitro | Anaerobe | 41 | 6,833333333 | 10.1016/j.anaerobe.2016.04.017 |
| BATISTA, AG; FERRARI, AS; DA CUNHA, DC; DA SILVA, JK; CAZARIN, CBB; CORREA, LC; PRADO, MA; DE CARVALHO-SILVA, LB; ESTEVES, EA; MAROSTICA, MR | 2016 | Polyphenols, antioxidants, and antimutagenic effects of copaifera langsdorffii fruit | *Copaifera langsdorffii* | Antimutagenic | In vitro | Food chemistry | 35 | 5,833333333 | 10.1016/j.foodchem.2015.11.093 |
| BORGES, CHG; CRUZ, MG; CARNEIRO, LJ; DA SILVA, JJM; BASTOS, JK; TAVARES, DC; DE OLIVEIRA, PF; RODRIGUES, V; VENEZIANI, RCS; PARREIRA, RLT; CARAMORI, GF; NAGURNIAK, GR; MAGALHAES, LG; AMBROSIO, SR | 2016 | Copaifera duckei oleoresin and its main nonvolatile terpenes: in vitro schistosomicidal properties | *Copaifera duckei* | Antiparasitic | In vitro | Chemistry & biodiversity | 21 | 3,5 | 10.1002/cbdv.201600065 |
| LEANDRO, LF; MORAES, TD; DE OLIVEIRA, PF; ALVES, JM; SENEDESE, JM; OZELIN, SD; RESENDE, FA; DE GRANDIS, RA; VARANDA, EA; BASTOS, JK; TAVARES, DC; MARTINS, CHG | 2016 | Assessment of the antibacterial, cytotoxic and mutagenic potential of the phenolic-rich hydroalcoholic extract from copaifera trapezifolia hayne leaves | *Copaifera trapezifolia Hayne* | Antibacterial, cytotoxic and mutagenic | In vitro | Journal of medical microbiology | 13 | 2,166666667 | 10.1099/jmm.0.000316 |
| MORAES, TD; LEANDRO, LF; SILVA, LD; SANTIAGO, MB; SOUZA, AB; FURTADO, RA; TAVARES, DC; VENEZIANI, RCS; AMBROSIO, SR; BASTOS, JK; MARTINS, CHG | 2016 | In vitro evaluation of copaifera oblongifolia oleoresin against bacteria causing oral infections and assessment of its cytotoxic potential | *Copaifera oblongifolia* | Antimicrobial | In vitro | Current pharmaceutical biotechnology | 12 | 2 | 10.2174/1389201017666160415155359 |
| BORGES, VRD; DA SILVA, JH; BARBOSA, SS; NASCIUTTI, LE; CABRAL, LM; DE SOUSA, VP | 2016 | Development and pharmacological evaluation of in vitro nanocarriers composed of lamellar silicates containing copaiba oil-resin for treatment of endometriosis | *Copaifera langsorffi* | Anti-endometriosis | In vitro | Materials science & engineering c-materials for biological applications | 11 | 1,833333333 | 10.1016/j.msec.2016.03.094 |
| TOBOUTI, PL; MUSSI, MCM; ROSSI, DCP; PIGATTI, FM; TABORDA, CP; TAVEIRA, LAD; DE SOUSA, SCOM | 2016 | Influence of melaleuca and copaiba oils on candida albicans adhesion | *Copaifera officinalis* | Antifungal | In vitro | Gerodontology | 10 | 1,666666667 | 10.1111/ger.12172 |
| SILVA, GO; CAVALCANTI, BN; OLIVEIRA, TR; BIN, CV; CAMARGO, SEA; CAMARGO, CHR | 2016 | Cytotoxicity and genotoxicity of natural resin-based experimental endodontic sealers | *Copaifera multijuga* | Biocompatible in resin sealers | In vitro | Clinical oral investigations | 7 | 1,166666667 | 10.1007/s00784-015-1567-4 |
| DE BARI, CC; SAMPAIO, F; CONDE, N; MOURA, L; VEIGA, V; BARBOSA, G; VASCONCELLOS, M; TODA, C; VENANCIO, G; BANDEIRA, MF | 2016 | Amazon emulsions as cavity cleansers: antibacterial activity, cytotoxicity and changes in human tooth color. | *Copaifera multijuga Hayne* | Antibacterial | In vitro | Revista brasileira de farmacognosia-brazilian journal of pharmacognosy | 4 | 0,666666667 | 10.1016/j.bjp.2016.03.010 |
| DOS SANTOS, ACV; FERNANDES, CC; LOPES, LM; DE SOUSA, AH | 2016 | Inseticidal oils from amazon plants in control of fall armyworm | *Copaifera spp* | Inseticidal | In vitro | Revista caatinga | 4 | 0,666666667 | Not informed |
| SAMIA, RR; DE OLIVEIRA, RL; MOSCARDINI, VF; CARVALHO, GA | 2016 | Effects of aqueous extracts of copaifera langsdorffii (fabaceae) on the growth and reproduction of spodoptera frugiperda (j. E. Smith) (lepidoptera: noctuidae) | *Copaifera langsdorffii* | Pesticide | In vitro | Neotropical entomology | 1 | 0,166666667 | 10.1007/s13744-016-0398-6 |
| MOTTA, EVS; LEMOS, M; COSTA, JC; BANDERO, VC; SASSE, A; SHERIDAN, H; BASTOS, JK | 2017 | Galloylquinic acid derivatives from copaifera langsdorffii leaves display gastroprotective activity | *Copaifera langsdorffii* | Gastroprotective | In vitro and in vivo | Chemico-biological interactions | 20 | 4 | 10.1016/j.cbi.2016.11.028 |
| GHIZONI, CVC; AMES, APA; LAMEIRA, OA; AMADO, CAB; NAKANISHI, ABS; BRACHT, L; NATALI, MRM; PERALTA, RM; BRACHT, A; COMAR, JF | 2017 | Anti-inflammatory and antioxidant actions of copaiba oil are related to liver cell modifications in arthritic rats | *Copaifera reticulata* | Anti-Inflammatory and Antioxidant | In vivo | Journal of cellular biochemistry | 19 | 3,8 | 10.1002/jcb.25998 |
| DA COSTA, JC; VALLADAO, GMR; PALA, G; GALLANI, SU; KOTZENT, S; CROTTI, AEM; FRACAROLLI, L; DA SILVA, JJM; PILARSKI, F | 2017 | Copaifera duckei oleoresin as a novel alternative for treatment of monogenean infections in pacu piaractus mesopotamicus | *Copaifera duckei* | Anthelmintic | In vitro | Aquaculture | 18 | 3,6 | 10.1016/j.aquaculture.2016.11.041 |
| TOBOUTI, PL; MARTINS, TCD; PEREIRA, TJ; MUSSI, MCM | 2017 | Antimicrobial activity of copaiba oil: a review and a call for further research | *Copaifera spp* | Antimicrobial | Review paper | Biomedicine & pharmacotherapy | 18 | 3,6 | 10.1016/j.biopha.2017.07.092 |
| MARANGON, CA; MARTINS, VCA; LEITE, PMF; SANTOS, DA; NITSCHKE, M; PLEPIS, AMG | 2017 | Chitosan/gelatin/copaiba oil emulsion formulation and its potential on controlling the growth of pathogenic bacteria |  | Antibacterial | In vitro | Industrial crops and products | 16 | 3,2 | 10.1016/j.indcrop.2017.02.007 |
| PASCOAL, DRC; CABRAL-ALBUQUERQUE, ECM; VELOZO, ES; DE SOUSA, HC; DE MELO, SABV; BRAGA, MEM | 2017 | Copaiba oil-loaded commercial wound dressings using supercritical co2: a potential alternative topical antileishmanial treatment | *Copaifera spp* | Antiparasitic | In vitro | Journal of supercritical fluids | 16 | 3,2 | 10.1016/j.supflu.2017.02.012 |
| PINHEIRO, JGD; TAVARES, ED; DA SILVA, SS; SILVA, JF; DE CARVALHO, YMBG; FERREIRA, MRA; ARAUJO, AAD; BARBOSA, EG; PEDROSA, MDF; SOARES, LAL; DE AZEVEDO, EP; DA VEIGA, VF; DE LIMA, AAN | 2017 | Inclusion complexes of copaiba (copaifera multijuga hayne) oleoresin and cyclodextrins: physicochemical characterization and anti-inflammatory activity | *Copaifera multijuga Hayne* | Anti-Inflammatory | In vitro and in vivo | International journal of molecular sciences | 14 | 2,8 | 10.3390/ijms18112388 |
| WAGNER, VP; WEBBER, LP; ORTIZ, L; RADOS, PV; MEURER, L; LAMEIRA, OA; LIMA, RR; MARTINS, MD | 2017 | Effects of copaiba oil topical administration on oral wound healing | *Copaifera reticulata Ducke* | Healing | In vivo | Phytotherapy research | 13 | 2,6 | 10.1002/ptr.5845 |
| PENIDO, AB; DE MORAIS, SM; RIBEIRO, AB; ALVES, DR; RODRIGUES, ALM; DOS SANTOS, LH; DE MENEZES, JESA | 2017 | Medicinal plants from northeastern brazil against alzheimer's disease | *Copaifera langsdorffii* | Against Alzheimer's Disease | In vitro | Evidence-based complementary and alternative medicine | 12 | 2,4 | 10.1155/2017/1753673 |
| TEIXEIRA, FB; SILVA, RD; LAMEIRA, OA; WEBBER, LP; COUTO, RSD; MARTINS, MD; LIMA, RR | 2017 | Copaiba oil-resin (copaifera reticulata ducke) modulates the inflammation in a model of injury to rats' tongues | *Copaifera reticulata Ducke* | Anti-inflammatory | In vivo | Bmc complementary and alternative medicine | 12 | 2,4 | 10.1186/s12906-017-1820-2 |
| DE SOUZA, GAG; DA SILVA, NC; DE SOUZA, J; DE OLIVEIRA, KRM; DA FONSECA, AL; BARATTO, LC; DE OLIVEIRA, ECP; VAROTTI, FD; MORAES, WP | 2017 | In vitro and in vivo antimalarial potential of oleoresin obtained from copaifera reticulata ducke (fabaceae) in the brazilian amazon rainforest | *Copaifera reticulata Ducke* | Antimalarial | In vitro and in vivo | Phytomedicine | 11 | 2,2 | 10.1016/j.phymed.2016.11.021 |
| DE ALBUQUERQUE, KCO; DA VEIGA, ADS; SILVA, JVDE; BRIGIDO, HPC; FERREIRA, EPD; COSTA, EVS; MARINHO, AMD; PERCARIO, S; DOLABELA, MF | 2017 | Brazilian amazon traditional medicine and the treatment of difficult to heal leishmaniasis wounds with copaifera | *Copaifera spp* | Antileishmanial and Healing | Review paper | Evidence-based complementary and alternative medicine | 10 | 2 | 10.1155/2017/8350320 |
| CAMPOS, C; DE CASTRO, AL; TAVARES, AMV; FERNANDES, RO; ORTIZ, VD; BARBOZA, TE; PEREIRA, C; APEL, M; DA SILVA, OS; LLESUY, S; ARAUJO, ASD; BELLO-KLEIN, A | 2017 | Effect of free and nanoencapsulated copaiba oil on monocrotaline-induced pulmonary arterial hypertension | *Copaifera spp* | Pulmonary effects | In vivo | Journal of cardiovascular pharmacology | 10 | 2 | Not informed |
| ALVES, JM; SENEDESE, JM; LEANDRO, LF; CASTRO, PT; PEREIRA, DE; CARNEIRO, LJ; AMBROSIO, SR; BASTOS, JK; TAVARES, DC | 2017 | Copaifera multijuga oleoresin and its constituent diterpene (-)-copalic acid: genotoxicity and chemoprevention study | *Copaiferamultijugaoleoresin* | Genotoxicityand the chemopreventivepotential | In vivo | Mutation research-genetic toxicology and environmental mutagenesis | 7 | 1,4 | 10.1016/j.mrgentox.2017.05.001 |
| OTAGUIRI, ES; MORGUETTE, AEB; BIASI-GARBIN, RP; MOREY, AT; LANCHEROS, CAC; KIAN, D; DE OLIVEIRA, AG; KERBAUY, G; PERUGINI, MRE; DURAN, N; NAKAMURA, CV; DA VEIGA, VF; NAKAZATO, G; PINGE, P; YAMAUCHI, LM; YAMADA-OGATTA, SF | 2017 | Antibacterial combination of oleoresin from copaifera multijuga hayne and biogenic silver nanoparticles towards streptococcus agalactiae | *Copaifera multijuga Hayne* | Antibacterial | In vitro | Current pharmaceutical biotechnology | 5 | 1 | 10.2174/1389201017666161213151919 |
| LUCCA, LG; DE MATOS, SP; DE MATTOS, CB; TEIXEIRA, HF; LIMBERGER, RP; VEIGA, VF; DE ARAUJO, BV; KOESTER, LS | 2017 | Nanoemulsification potentiates in vivo antiedematogenic effect of copaiba oil | *Copaifera multijuga* | Antiedematogenic | In vivo | Journal of biomedical nanotechnology | 5 | 1 | 10.1166/jbn.2017.2366 |
| NAKAMURA, MT; ENDO, EH; DE SOUSA, JPB; CALLEJON, DR; UEDA-NAKAMURA, T; DIAS, BP; DE FREITAS, O; NAKAMURA, CV; LOPES, NP | 2017 | Copaiba oil and its constituent copalic acid as chemotherapeutic agents against dermatophytes | *Copaifera spp* | Antifungal | In vitro | Journal of the brazilian chemical society | 5 | 1 | 10.21577/0103-5053.20160309 |
| SVETLICHNY, G; KULKAMP-GUERREIRO, IC; LANA, DFD; BIANCHIN, MD; POHLMANN, AR; FUENTEFRIA, AM; GUTERRES, SS | 2017 | Assessing the performance of copaiba oil and allantoin nanoparticles on multidrug-resistant candida parapsilosis | *Copaifera spp* | Antifungal | In vitro | Journal of drug delivery science and technology | 4 | 0,8 | 10.1016/j.jddst.2017.05.020 |
| DIAS, FGG; JORGE, AT; PEREIRA, LD; FURTADO, RA; AMBROSIO, SR; BASTOS, JK; RAMOS, SB; CHAHUD, F; DIAS, LGGG; HONSHO, CD; TAVARES, DC | 2017 | Use of copaifera multijuga for acute corneal repair after chemical injury: a clinical, histopathological and toxicogenetic study | *Copaifera multijuga* | Corneal repair | In vivo | Biomedicine & pharmacotherapy | 2 | 0,4 | 10.1016/j.biopha.2017.11.099 |
| ZORTEA, T; BARETTA, D; VOLPATO, A; LORENZETTI, WR; SEGAT, JC; MACCARI, AP; SANTOS, RC; VAUCHER, RA; STEFANI, LM; DA SILVA, AS | 2017 | Repellent effects of andiroba and copaiba oils against musca domestica (common house fly) and ecotoxicological effects on the environment | *Copaifera reticulata* | Repellent | In vivo | Acta scientiae veterinariae | 2 | 0,4 | Not informed |
| LUCAS, FD; KANDROTAS, AL; NETO, EN; DE SIQUEIRA, CE; ANDRE, GS; BROMERSCHENKEL, I; PERRI, SHV | 2017 | Copaiba oil in experimental wound healing in horses | *Copaifera spp* | Healing | In vivo | Ciencia rural | 1 | 0,2 | 10.1590/0103-8478cr20151292 |
| LIMA, CS; SILVA, UDDE; GOES, LDM; HYACIENTH, BMD; CARVALHO, HD; FERNANDES, CP; CASTRO, AN; CARVALHO, JCT | 2017 | Non-clinical toxicity study of the oil-resin and vaginal cream of copaiba (copaifera duckei, dwyer) | *Copaifera duckei Dwyer* | Toxicity | In vivo | Cogent biology | 1 | 0,2 | 10.1080/23312025.2017.1394510 |
| HORACIO, BO; GERON, VLMG; FAVERO, MT; SPERETTA, G; MENEZES, MF | 2017 | Anti-inflammatory action of copaiba oil: possible contribution in the treatment of metabolic syndrome | *Copaifera spp* | Anti-inflammatory | Review paper | Revista cientifica da faculdade de educacao e meio ambiente | 0 | 0 | Not informed |
| FARIA, AM; SANTOS, AD; FERREIRA, LL; BASTOS, TSA; LOULY, CCB; MATOS, MPC; DA CONCEICAO, EC; DE MOURA, VMBD | 2017 | Acaricidal activity of ethanolic extracts of stryphnodendron adstringens and lafoensia pacari and oil resins from copaifera sp and pterodon emarginatus against sarcoptes scabiei var. Suis | *Copaifera spp* | Antimicrobial and Acaricidal | In vitro | Pesquisa veterinaria brasileira | 0 | 0 | 10.1590/S0100-736X2017001200009 |
| DA TRINDADE, R; DA SILVA, JK; SETZER, WN | 2018 | Copaifera of the neotropics: a review of the phytochemistry and pharmacology | *Copaifera spp* | Antimicrobial, Anti-inflammatory, Gastroprotective, Healing, Antioxidant, antitumor | Review paper | International journal of molecular sciences | 46 | 11,5 | 10.3390/ijms19051511 |
| DE MORAES, ARDP; TAVARES, GD; ROCHA, FJS; DE PAULA, E; GIORGIO, S | 2018 | Effects of nanoemulsions prepared with essential oils of copaiba- and andiroba against leishmania infantum and leishmania amazonensis infections | *Copaifera spp* | Antileishmanial | In vitro and in vivo | Experimental parasitology | 20 | 5 | 10.1016/j.exppara.2018.03.005 |
| DA SILVA, BJM; HAGE, AAP; SILVA, EO; RODRIGUES, APD | 2018 | Medicinal plants from the brazilian amazonian region and their antileishmanial activity: a review | *Copaifera spp* | Antileishmanial | Review paper | Journal of integrative medicine-jim | 20 | 5 | 10.1016/j.joim.2018.04.004 |
| DIEFENBACH, AL; MUNIZ, FWMG; OBALLE, HJR; ROSING, CK | 2018 | Antimicrobial activity of copaiba oil (copaifera ssp.) On oral pathogens: systematic review | *Copaifera spp* | Antimicrobial | Review paper | Phytotherapy research | 18 | 4,5 | 10.1002/ptr.5992 |
| LUCCA, LG; DE MATOS, SP; KREUTZ, T; TEIXEIRA, HF; VEIGA, VF; DE ARAUJO, BV; LIMBERGER, RP; KOESTER, LS | 2018 | Anti-inflammatory effect from a hydrogel containing nanoemulsified copaiba oil (copaifera multijuga hayne) | *Copaifera multijuga Hayne* | Anti-inflammatory | In vivo | Aaps pharmscitech | 16 | 4 | 10.1208/s12249-017-0862-6 |
| FURTADO, RA; DE OLIVEIRA, PF; SENEDESE, JM; OZELIN, SD; DE SOUZ, LDR; LEANDRO, LF; DE OLIVEIRA, WL; DA SILVA, JJM; OLIVEIRA, LC; ROGEZ, H; AMBROSIO, SR; VENEZIANI, RCS; BASTOS, JK; TAVARES, DC | 2018 | Assessment of genotoxic activity of oleoresins and leaves extracts of six copaifera species for prediction of potential human risks | *C. Duckei, C. Multijuga, C. Paupera, C. Pubiflora, C. Reticulata and C. Trapezifolia* | Genotoxicity | In vitro | Journal of ethnopharmacology | 15 | 3,75 | 10.1016/j.jep.2018.04.002 |
| CAMPOS-CARRARO, C; TURCK, P; DE LIMA-SEOLIN, BG; TAVARES, AMV; LACERDA, DD; CORSSAC, GB; TEIXEIRA, RB; HICKMANN, A; LLESUY, S; ARAUJO, ASD; BELLO-KLEIN, A | 2018 | Copaiba oil attenuates right ventricular remodeling by decreasing myocardial apoptotic signaling in monocrotaline-induced rats | *Copaifera spp* | Cardioprotective | In vivo | Journal of cardiovascular pharmacology | 10 | 2,5 | 10.1097/FJC.0000000000000617 |
| CHURQUI, MP; LIND, L; THORN, K; SVENSSON, A; SAVOLAINEN, O; ARANDA, KT; ERIKSSON, K | 2018 | Extracts of equisetum giganteum l and copaifera reticulate ducke show strong antiviral activity against the sexually transmitted pathogen herpes simplex virus type 2 | *Copaifera reticulata Ducke* | Antiviral | In vitro and in vivo | Journal of ethnopharmacology | 9 | 2,25 | 10.1016/j.jep.2017.08.010 |
| VIEIRA, RGL; MORAES, TD; SILVA, LD; BIANCHI, TC; VENEZIANI, RCS; AMBROSIO, SR; BASTOS, JK; PIRES, RH; MARTINS, CHG | 2018 | In vitro studies of the antibacterial activity of copaifera spp. Oleoresins, sodium hypochlorite, and peracetic acid against clinical and environmental isolates recovered from a hemodialysis unit | *Copaifera duckei, C. Reticulata, and C. Oblongifolia* | Antibacterial | In vitro | Antimicrobial resistance and infection control | 8 | 2 | 10.1186/s13756-018-0307-3 |
| PAVAN, E; DAMAZO, AS; LEMOS, LMS; ADZU, B; BALOGUN, SO; ARUNACHALAM, K; MARTINS, DTD | 2018 | Evaluation of genotoxicity and subchronic toxicity of the standardized leaves infusion extract of copaifera malmei harms in experimental models | *Copaifera malmei Harms* | Antigenotoxicity | In vivo | Journal of ethnopharmacology | 8 | 2 | 10.1016/j.jep.2017.09.027 |
| ALVES, JM; LEANDRO, LF; SENEDESE, JM; DE CASTRO, PT; PEREIRA, DE; RESENDE, FA; CAMPOS, DL; DA SILVA, JJM; VARANDA, EA; BASTOS, JK; AMBROSIO, SR; TAVARES, DC | 2018 | Antigenotoxicity properties of copaifera multijuga oleoresin and its chemical marker, the diterpene (-)-copalic acid | *Copaifera multijuga* | Antigenotoxicity | In vitro and in vivo | Journal of toxicology and environmental health-part a-current issues | 8 | 2 | 10.1080/15287394.2017.1420505 |
| BAHR, T; ALLRED, K; MARTINEZ, D; RODRIGUEZ, D; WINTERTON, P | 2018 | Effects of a massage-like essential oil application procedure using copaiba and deep blue oils in individuals with hand arthritis | *Copaifera spp* | Analgesic and anti-inflammatory | Human reports | Complementary therapies in clinical practice | 7 | 1,75 | 10.1016/j.ctcp.2018.10.004 |
| ABRAO, F; ALVES, JA; ANDRADE, G; DE OLIVEIRA, PF; AMBROSIO, SR; VENEZIANI, RCS; TAVARES, DC; BASTOS, JK; MARTINS, CHG | 2018 | Antibacterial effect of copaifera duckei dwyer oleoresin and its main diterpenes against oral pathogens and their cytotoxic effect | *Copaifera duckei Dwyer* | Antibacterial | In vitro | Frontiers in microbiology | 7 | 1,75 | 10.3389/fmicb.2018.00201 |
| KIAN, D; LANCHEROS, CAC; ASSOLINI, JP; ARAKAWA, NS; VEIGA, VF; NAKAMURA, CV; PINGE, P; CONCHON-COSTA, I; PAVANELLI, WR; YAMADA-OGATTA, SF; YAMAUCHI, LM | 2018 | Trypanocidal activity of copaiba oil and kaurenoic acid does not depend on macrophage killing machinery | *Copaifera martii* | Anti-trypanossomal | In vivo | Biomedicine & pharmacotherapy | 7 | 1,75 | 10.1016/j.biopha.2018.04.164 |
| MACEDO, MJF; RIBEIRO, DA; SANTOS, MD; DE MACEDO, DG; MACEDO, JGF; DE ALMEIDA, BV; SARAIVA, ME; DE LACERDA, MNS; SOUZA, MMD | 2018 | Fabaceae medicinal flora with therapeutic potential in savanna areas in the chapada do araripe, northeastern brazil | *Copaifera langsdorffii* | Anti rheumatism, gastric problems and general pains | Human reports | Revista brasileira de farmacognosia-brazilian journal of pharmacognosy | 7 | 1,75 | 10.1016/j.bjp.2018.06.010 |
| BARBOSA, MMC; VICENTINI, FA; CASTRO-GHIZONI, CV; LAMEIRA, OA; SA-NAKANISHI, AB; BRACHT, L; PERALTA, RM; NATALI, MRM; BRACHT, A; COMAR, JF | 2018 | Copaiba oil decreases oxidative stress and inflammation but not colon damage in rats with tnbs-induced colitis | *Copaifera reticulata Ducke* | Anti-inflammatory and antioxidant | In vivo | Endocrine metabolic & immune disorders-drug targets | 6 | 1,5 | 10.2174/1871530318666180215102029 |
| VALENTIM, DSS; DUARTE, JL; OLIVEIRA, AEMFM; CRUZ, RAS; CARVALHO, JCT; SOLANS, C; FERNANDES, CP; TAVARES-DIAS, M | 2018 | Effects of a nanoemulsion with copaifera officinalis oleoresin against monogenean parasites of colossoma macropomum: a neotropical serrasalmidae | *Copaifera officinalis* | Antiparasitic | In vivo | Journal of fish diseases | 6 | 1,5 | 10.1111/jfd.12793 |
| FERNANDEZ, YA; DAMASCENO, JL; ABRAO, F; SILVA, TD; CANDIDO, ADP; FREGONEZI, NF; RESENDE, FA; RAMOS, SB; AMBROSIO, SR; VENEZIANI, RCS; BASTOS, JK; MARTINS, CHG | 2018 | Antibacterial, preservative, and mutagenic potential of copaifera spp. Oleoresins against causative agents of foodborne diseases | *C. Duckei, C. Reticulata, C. Paupera, and C. Pubiflora* | Antibacterial | In vitro | Foodborne pathogens and disease | 4 | 1 | 10.1089/fpd.2018.2478 |
| CARVALHO, HO; DOS SANTOS, IVF; DA ROCHA, CF; BARROS, ASA; SOUZA, BSFE; FERREIRA, IM; BEZERRA, RM; LIMA, CS; CASTRO, AN; CARVALHO, JCT | 2018 | Effect of the treatment of copaifera duckei oleoresin (copaiba) in streptozotocin-induced diabetic rats | *Copaifera duckei* | Antidiabetic | In vivo | Revista brasileira de farmacognosia-brazilian journal of pharmacognosy | 3 | 0,75 | 10.1016/j.bjp.2018.09.004 |
| QUINONES, OG; HOSSY, BH; PADUA, TA; MIGUEL, NCD; ROSAS, EC; RAMOS, MFD; PIERRE, MBR | 2018 | Copaiba oil enhances in vitro/in vivo cutaneous permeability and in vivo anti-inflammatory effect of celecoxib | *Copaifera spp* | Anti-inflammatory | In vitro and in vivo | Journal of pharmacy and pharmacology | 3 | 0,75 | 10.1111/jphp.12906 |
| FEITOSA, DJS; DE CARVALHO, LTF; ROCHA, IRD; DE BRITO, CN; MOREIRA, RA; DE BARROS, CAV | 2018 | Effects of copaiba oil in the healing process of urinary bladder in rats | *Copaifera reticulata* | Healing | In vivo | International braz j urol | 3 | 0,75 | 10.1590/S1677-5538.IBJU.2017.0143 |
| QUINONES, OG; ABRANCHES, RP; NAKAMURA, MJ; RAMOS, MFD; PIERRE, MBR | 2018 | Copaiba oil: chemical composition and influence on in-vitro cutaneous permeability of celecoxib | *Copaifera-multijuga hayne* | ANTI-INFLAMMATORY | In vitro | Current drug delivery | 0 | 0 | 10.2174/1567201814666170825154453 |
| ARRUDA, C; MEJIA, JAA; RIBEIRO, VP; BORGES, CHG; MARTINS, CHG; VENEZIANI, RCS; AMBROSIO, SR; BASTOS, JK | 2019 | Occurrence, chemical composition, biological activities and analytical methods on copaifera genus-a review | *Copaifera spp* | Anti-inflammatory, antimicrobial, antiparasitic, gastroprotective, antitumor | Review paper | Biomedicine & pharmacotherapy | 30 | 10 | 10.1016/j.biopha.2018.10.030 |
| DEBONE, HS; LOPES, PS; SEVERINO, P; YOSHIDA, CMP; SOUTO, EB; DA SILVA, CF | 2019 | Chitosan/copaiba oleoresin films for would dressing application | *Copaifera reticulata duckey* | Healing | In vitro | International journal of pharmaceutics | 19 | 6,333333333 | 10.1016/j.ijpharm.2018.11.054 |
| BARBOSA, ALP; WENZEL-STORJOHANN, A; BARBOSA, JD; ZIDORN, C; PEIFER, C; TASDEMIR, D; CICEK, SS | 2019 | Antimicrobial and cytotoxic effects of the copaifera reticulata oleoresin and its main diterpene acids | *Copaifera reticulata duckey* | Antimicrobial | In vitro | Journal of ethnopharmacology | 17 | 5,666666667 | 10.1016/j.jep.2018.11.029 |
| GURGEL, ESC; DE OLIVEIRA, MS; SOUZA, MC; DA SILVA, SG; DE MENDONCA, MS; SOUZA, APD | 2019 | Chemical compositions and herbicidal (phytotoxic) activity of essential oils of three copaifera species (leguminosae-caesalpinoideae) from amazon-brazil | *Copaifera duckei (Dwyer), Copaifera martii (Hayne), and Copaifera reticulata (Ducke)* | Phytotoxic | In vitro | Industrial crops and products | 16 | 5,333333333 | 10.1016/j.indcrop.2019.111850 |
| SENEDESE, JM; RINALDI-NETO, F; FURTADO, RA; NICOLLELA, HD; DE SOUZA, LDR; RIBEIRO, AB; FERREIRA, LS; MAGALHAES, GM; CARLOS, IZ; DA SILVA, JJM; TAVARES, DC; BASTOS, JK | 2019 | Chemopreventive role of copaifera reticulata ducke oleoresin in colon carcinogenesis | *Copaifera reticulata Ducke* | Anticancer | In vivo | Biomedicine & pharmacotherapy | 9 | 3 | 10.1016/j.biopha.2018.12.091 |
| ALOKE, C; IBIAM, UA; OBASI, NA; ORJI, OU; EZEANI, NN; AJA, PM; ALUM, EU; MORDI, JC | 2019 | Effect of ethanol and aqueous extracts of seed pod of copaifera salikounda (heckel) on complete freund's adjuvant-induced rheumatoid arthritis in rats | *Copaifera salikounda* | Antirheumatoid arthritis | In vivo | Journal of food biochemistry | 7 | 2,333333333 | 10.1111/jfbc.12912 |
| DALENOGARE, DP; FERRO, PR; DE PRA, SDT; RIGO, FK; ANTONIAZZI, CTD; DE ALMEIDA, AS; DAMIANI, AP; STRAPAZZON, G; SARDINHA, TTD; GALVANI, NC; BOLIGON, AA; DE ANDRADE, VM; BRUM, ED; OLIVEIRA, SM; TREVISAN, G | 2019 | Antinociceptive activity of copaifera officinalis jacq. L oil and kaurenoic acid in mice | *Copaifera officinalis Jacq.* | Antinociceptive | In vivo | Inflammopharmacology | 5 | 1,666666667 | 10.1007/s10787-019-00588-3 |
| DE SOUSA, SF; PAES, JB; ARANTES, MDC; LOPES, DV; NICACIO, MA | 2019 | Efficiency of vegetable oils in wood resistance to cryptotermes brevis termites | *Copaifera langsdorffii* | Biological resistance of wood to dry-wood termites | In vitro | Floresta e ambiente | 4 | 1,333333333 | 10.1590/2179-8087.078017 |
| DA CUNHA, APS; BALDISSERA, L; PEREIRA, DL; ALBIERO, LR; CASTOLDI, L; SINHORIN, AP; SINHORIN, VDG | 2019 | Evaluation of the antioxidant potential of copaifera multijuga in ehrlich tumor-bearing mice | *Copaifera multijuga* | Antioxidant | In vivo | Acta amazonica | 3 | 1 | 10.1590/1809-4392201800672 |
| SILVA, S; ALVES, N; SILVA, P; VIEIRA, T; MACIEL, P; CASTELLANO, LR; BONAN, P; VELOZO, C; ALBUQUERQUE, D | 2019 | Antibacterial activity of rosmarinus officinalis, zingiber officinale, citrus aurantium bergamia, and copaifera officinalis alone and in combination with calcium hydroxide against enterococcus faecalis | *Copaifera officinalis* | Antibacterial | In vitro | Biomed research international | 2 | 0,666666667 | 10.1155/2019/8129439 |
| TEIXEIRA, RKC; COSTA, FLD; CALVO, FC; DOS SANTOS, DR; YASOJIMA, EY; BRITO, MVH | 2019 | Effect of copaiba oil in intestinal mucosa of rats submitted to hypovolemic shock | *Copaifera officinalis* | Hypovolemic shock treatment | In vivo | Abcd-arquivos brasileiros de cirurgia digestiva-brazilian archives of digestive surgery | 2 | 0,666666667 | 10.1590/0102-672020190001e1451 |
| MORGUETTE, AEB; BIGOTTO, BG; VARELLA, RD; ANDRIANI, GM; SPOLADORI, LFD; PEREIRA, PML; DE ANDRADE, FG; LANCHEROS, CAC; NAKAMURA, CV; ARAKAWA, NS; BRUSCHI, ML; TOMAZ, JC; LONNI, AASG; KERBAUY, G; TAVARES, ER; YAMAUCHI, LM; YAMADA-OGATTA, SF | 2019 | Hydrogel containing oleoresin from copaifera officinalis presents antibacterial activity against streptococcus agalactiae | *Copaifera officinalis* | Antibacterial | In vitro | Frontiers in microbiology | 1 | 0,333333333 | 10.3389/fmicb.2019.02806 |
| FERNANDES, EG; VALERIO, HM; DUARTE, KLR; CAPUCHINHO, LMD; FAGUNDES, M | 2019 | Fungi associated with copaifera oblongifolia (fabaceae) seeds: occurrence and possible effects on seed germination | *Copaifera oblongifolia* | Antifungal | In vitro | Acta botanica brasilica | 1 | 0,333333333 | 10.1590/0102-33062018abb0100 |
| SILVA, AA; SANTOS, IF; ANJOS, MM; PASCOLI, IC; RUIZ, SP; MIKEHA, JMG; MACHINSKI, M; NAKAMURA, TU; NAKAMURA, CV; ABREU, FBA | 2019 | Evaluation of the antibacterial and sporicidal activity of the essential oils of copaifera multijuga and thymus vulgaris against alicyclobacillus acidoterrestris | *Copaifera multijuga* | Antibacterial | In vitro | International food research journal | 0 | 0 | Not informed |
| BARBOSA, ALP; WENZEL-STORJOHANN, A; BARBOSA, JD; ZIDORN, C; PEIFER, C; TASDEMIR, D; CICEK, SS | 2019 | Antimicrobial and cytotoxic properties of the copaifera reticulata oleoresin and its major diterpene acids | *Copaifera reticulata* | Antimicrobial | In vitro | Planta medica | 0 | 0 | 10.1055/s-0039-3400134 |
| RIBEIRO, MF; DE OLIVEIRA, FL; SOUZA, AM; MACHADO, TD; CARDOSO, PF; SOBRINHO, AP; NASCIMENTO, AS; DE SOUZA, CMV; ELIAS, SC | 2019 | Effects of copaiba oil on dermonecrosis induced by loxosceles intermedia venom | *Copaifera spp* | Healing | In vivo | Journal of venomous animals and toxins including tropical diseases | 0 | 0 | 10.1590/1678-9199-JVATITD-1493-18 |
| RODRIGUES, PFG; ALBIERO, LR; NERY, EF; KELLY, TO; DALAZEN, JCS; PEREIRA, DL; SINHORIN, AP; SINHORIN, VDG; CASTOLDI, L | 2019 | Natural killer activity of the spleen cells of ehrlich tumor-bearing mice treated with copaifera multijuga extract | *Copaifera multijuga* | Immunomodulatory | In vivo | Scientia medica | 0 | 0 | 10.15448/1980-6108.2019.1.32408 |
| GOVERNA, P; BIAGI, M | 2020 | Copaifera langsdorffii desf.: in vitro investigation on anti-helicobacter pylori and anti-inflammatory activities of oleoresin and fruit methanolic extract | *Copaifera langsdorffii* | Anti-bacterial and anti-inflammatory | In vitro | Plant biosystems | 7 | 3,5 | 10.1080/11263504.2019.1578284 |
| ALDANA, JA; DE GRANDIS, RA; NICOLELLA, H; GUISSONI, APP; SQUARISI, I; ARRUDA, C; RIBEIRO, VP; TAVARES, DC; BARCELOS, GRM; ANTUNES, LMG; BASTOS, JK | 2020 | Evaluation of cytoprotective effects of compoundsisolated fromcopaifera langsdorffiidesf. Against inducedcytotoxicity by exposure to methylmercury and lead | *Copaifera langsdorffii* | Cytoprotective | In vitro | Natural product research | 5 | 2,5 | 10.1080/14786419.2018.1543673 |
| URASAKI, Y; BEAUMONT, C; WORKMAN, M; TALBOT, JN; HILL, DK; LE, TT | 2020 | Fast-acting and receptor-mediated regulation of neuronal signaling pathways by copaiba essential oil | *Copaifera reticulata, Copaifera officinalis, Copaifera coriacea, and Copaifera langsdorffii* | Effects on neuronal signaling pathways | In vitro | International journal of molecular sciences | 5 | 2,5 | 10.3390/ijms21072259 |
| NIGRO, F; CERQUEIRA, C; ROSSI, A; CARDOSO, V; VERMELHO, AB; RICCI, E; DOS SANTOS, EP; MANSUR, CRE | 2020 | Development, characterization and in vitro toxicity evaluation of nanoemulsion-loaded hydrogel based on copaiba oil and coenzyme q10 | *Copaifera spp* | Toxicity | In vitro | Colloids and surfaces a-physicochemical and engineering aspects | 5 | 2,5 | 10.1016/j.colsurfa.2019.124132 |
| URASAKI, Y; BEAUMONT, C; TALBOT, JN; HILL, DK; LE, TT | 2020 | Akt3 regulates the tissue-specific response to copaiba essential oil | *Copaifera spp* | Akt3 expression | In vitro | International journal of molecular sciences | 4 | 2 | 10.3390/ijms21082851 |
| MALHEIROS, DF; SARQUIS, IR; FERREIRA, IM; MATHEWS, PD; MERTINS, O; TAVARES-DIAS, M | 2020 | Nanoemulsions with oleoresin of copaifera reticulata (leguminosae) improve anthelmintic efficacy in the control of monogenean parasites when compared to oleoresin without nanoformulation | *Copaifera reticulata* | Anthelmintic | In vitro | Journal of fish diseases | 4 | 2 | 10.1111/jfd.13168 |
| SILVA, LD; MIRANDA, MACM; DE FREITAS, JV; FERREIRA, SFA; LIMA, ECD; DE OLIVEIRA, CMA; KATO, L; TEREZAN, AP; RODRIGUEZ, AFR; FARIA, FSEDV; SOARES, CMD; PEREIRA, M | 2020 | Antifungal activity of copaiba resin oil in solution and nanoemulsion against paracoccidioides spp. | *Copaifera langsdorffii* | Antifungal | In vitro | Brazilian journal of microbiology | 4 | 2 | 10.1007/s42770-019-00201-3 |
| ALVARENGA, MOP; BITTENCOURT, LO; MENDES, PFS; RIBEIRO, JT; LAMEIRA, OA; MONTEIRO, MC; BARBOZA, CAG; MARTINS, MD; LIMA, RR | 2020 | Safety and effectiveness of copaiba oleoresin (c. Reticulata ducke) on inflammation and tissue repair of oral wounds in rats | *Copaifera reticulata Ducke* | Anti-inflammatory and healing | In vivo | International journal of molecular sciences | 4 | 2 | Not informed |
| DA SILVA, CED; OYAMA, J; FERREIRA, FBP; LALUCCI-SILVA, MPD; LORDANI, TVA; DA SILVA, RCD; MONICH, MDT; TEIXEIRA, JJV; LONARDONI, MVC | 2020 | Effect of essential oils on leishmania amazonensis: a systematic review | *Copaifera spp* | Antileishmanial | Review paper | Parasitology | 4 | 2 | 10.1017/S0031182020001304 |
| ANDRADE, G; ORLANDO, HCS; SCORZONI, L; PEDROSO, RS; ABRAO, F; CARVALHO, MTM; VENEZIANI, RCS; AMBROSIO, SR; BASTOS, JK; MENDES-GIANNINI, MJS; MARTINS, CHG; PIRES, RH | 2020 | Braziliancopaiferaspecies: antifungal activity against clinically relevantcandidaspecies, cellular target, and in vivo toxicity | *C. Duckei, C. Langsdorffii, C. Paupera, C. Reticulata, C. Trapezifolia, and C. Pubiflora* | Antifungal | In vitro | Journal of fungi | 3 | 1,5 | 10.3390/jof6030153 |
| RODRIGUES, GD; FILGUEIRAS, CT; GARCIA, VAD; DE CARVALHO, RA; VELASCO, JI; FAKHOURI, FM | 2020 | Antimicrobial activity and gc-ms profile of copaiba oil for incorporation into xanthosoma mafaffa schott starch-based films | *Copaifera spp* | Antimicrobial | In vitro | Polymers | 3 | 1,5 | 10.3390/polym12122883 |
| MORAES, TD; LEANDRO, LF; SANTIAGO, MB; SILVA, LD; BIANCHI, TC; VENEZIANI, RCS; AMBROSIO, SR; RAMOS, SB; BASTOS, JK; MARTINS, CHG | 2020 | Assessment of the antibacterial, antivirulence, and action mechanism of copaifera pubiflora oleoresin and isolated compounds against oral bacteria | *Copaifera pubiflora* | Antimicrobial | In vitro | Biomedicine & pharmacotherapy | 3 | 1,5 | 10.1016/j.biopha.2020.110467 |
| BECKER, G; BRUSCO, I; CASOTI, R; MARCHIORI, MCL; CRUZ, L; TREVISAN, G; OLIVEIRA, SM | 2020 | Copaiba oleoresin has topical antinociceptive activity in a uvb radiation-induced skin-burn model in mice | *Copaifera officinalis* | Antinociceptive | In vivo | Journal of ethnopharmacology | 3 | 1,5 | 10.1016/j.jep.2019.112476 |
| TEIXEIRA, SC; DE SOUZA, G; BORGES, BC; DE ARAUJO, TE; ROSINI, AM; AGUILA, FA; AMBROSIO, SR; VENEZIANI, RCS; BASTOS, JK; SILVA, MJB; MARTINS, CHG; BARBOSA, BD; FERRO, EAV | 2020 | Copaifera spp. Oleoresins impair toxoplasma gondii infection in both human trophoblastic cells and human placental explants | *C. Reticulata, C. Duckei, C. Paupera e C. Pubiflora* | Antiparasitic | In vitro | Scientific reports | 3 | 1,5 | 10.1038/s41598-020-72230-0 |
| DE SEIXAS, AT; GALLANI, SU; NORONHA, LD; SILVA, JJM; PASCHOAL, JAR; BASTOS, JK; VALLADAO, GMR | 2020 | Copaiferaoleoresins as a novel natural product against acanthocephalan in aquaculture: insights in the mode of action and toxicity | *Copaifera duckei, Copaifera pubiflora, Copaifera reticulata, Copaifera paupera and Copaifera multijuga* | Antiparasitic | In vitro | Aquaculture research | 3 | 1,5 | 10.1111/are.14813 |
| DA SILVA, YF; QUEIROZ, VD; KLING, ICS; ARCHANJO, BS; OLIVEIRA, RN; SIMAO, RA | 2020 | Antibacterial coatings on vegetable ivory obtained by cold plasma jet activation of silicone and copaiba oils | *Copaifera spp* | Antibacterial | In vitro | Plasma processes and polymers | 2 | 1 | 10.1002/ppap.202000035 |
| SILVA, TC; ARAUJO, ECG; LINS, TRD; REIS, CA; SANQUETTA, CR; DA ROCHA, MP | 2020 | Non-timber forest products in brazil: a bibliometric and a state of the art review | *Copaifera spp* | Anti-inflammatory | Review paper | Sustainability | 2 | 1 | 10.3390/su12177151 |
| CAPUTO, LS; CAMPOS, MIC; DIAS, HJ; CROTTI, AEM; FAJARDO, JB; VANELLI, CP; PRESTO, ACD; ALVES, MS; AARESTRUP, FM; PAULA, ACC; DA SILVA, AA; AARESTRUP, BJV; PEREIRA, OS; CORREA, JOD | 2020 | Copaiba oil suppresses inflammation in asthmatic lungs of balb/c mice induced with ovalbumin | *Copaifera spp* | Anti-inflammatory | In vivo | International immunopharmacology | 2 | 1 | 10.1016/j.intimp.2019.106177 |
| BANDEIRA, MFCL; FREITAS, AL; MENEZES, MDC; SILVA, JD; SOMBRA, GAD; ARAUJO, EAM; TODA, C; MORESCHI, ARC; CONDE, NCD | 2020 | Adhesive resistance of a copaiba oil-based dentin biomodifier | *Copaifera multijuga* | Dentin cleaning substance on microleakage and microtensile adhesive strength | In vitro | Brazilian oral research | 2 | 1 | 10.1590/1807-3107bor-2020.vol34.0001 |
| ALVES, JA; ABRAO, F; MORAES, TD; DAMASCENO, JL; MORAES, MFD; VENEZIANI, RCS; AMBROSIO, SR; BASTOS, JK; MIRANDA, MLD; MARTINS, CHG | 2020 | Investigation of copaifera genus as a new source of antimycobaterial agents | *C. Duckei, Chionanthus lucens ,C. Langsdorffii, C. Duckei, C. Reticulata, Copaifera oblongifolia, C. Trapezifolia and Copaifera multijuga* | Antimycobacterial | In vitro | Future science oa | 1 | 0,5 | 10.2144/fsoa-2020-0018 |
| BOMJARDIM, HA; OLIVEIRA, MC; BRITO, MF; OLIVEIRA, CMC; MONTEIRO, BM; SILVEIRA, NSS; BARBOSA, JD | 2020 | Bovine digital dermatitis in the brazilian amazon biome and topical treatment with copaifera reticulata oil | *Copaifera reticulata* | Antidermatiogenic | In vitro | Pesquisa veterinaria brasileira | 0 | 0 | 10.1590/1678-5150-PVB-6715 |
| DE SOUZA, SF; PAES, JB; ARANTES, MDC; DA SILVA, LF; DUDECKI, L | 2020 | Efficiency of andiroba, copaiba and jatropha oils to improve the resistance of pinus elliottii wood to wood-decay fungi | *Copaifera spp* | Antifungal | In vitro | Revista arvore | 0 | 0 | 10.1590/1806-908820200000030 |
| DE OLIVEIRA, DFF; NASCIMENTO, TP; RODRIGUES, CH; BATISTA, JMS; LIU, TPSL; DE MEDEIROS, ES; MOTA, RA; COSTA, RMPB; PORTO, TS; PORTO, CS; PORTO, ALF | 2020 | Antimicrobial potential of copaiba oil (copaifera multijuga hayne-leguminosae) against bubaline mastitis multiresistant isolates | *Copaifera multijuga Hayne* | Antimicrobial | In vitro | Anais da academia brasileira de ciencias | 0 | 0 | 10.1590/0001-3765202020200521 |
| SANTOS, DG; CASTRO, VS; JUNIOR, CAC; PINTO, AC; UEKANE, TM; REZENDE, CM | 2020 | Copaifera reticulata: chemical characterization and bactericidal activity against pathogens in foods | *Copaifera Reticulata* | Antioxidant | In vivo | Revista virtual de quimica | 0 | 0 | 10.21577/1984-6835.20200038 |
| SIMARO, GV; LEMOS, M; DA SILVA, JJM; RIBEIRO, VP; ARRUDA, C; SCHNEIDER, AH; WANDERLEY, CWD; CARNEIRO, LJ; MARIANO, RL; AMBROSIO, SR; DE ANDRADE, SF; BANDERO, VC; SASSE, A; SHERIDAN, H; SILVA, MLAE; BASTOS, JK | 2021 | Antinociceptive and anti-inflammatory activities of copaifera pubiflora benth oleoresin and its major metabolite ent-hardwickiic acid | *Copaifera pubiflora Benth* | Antinociceptive and anti-inflammatory | In vitro and in vivo | Journal of ethnopharmacology | 5 | 5 | 10.1016/j.jep.2021.113883 |
| ABRAO, F; SILVA, TS; MOURA, CL; AMBROSIO, SR; VENEZIANI, RCS; DE PAIVA, REF; BASTOS, JK; MARTINS, CHG | 2021 | Oleoresins and naturally occurring compounds of copaifera genus as antibacterial and antivirulence agents against periodontal pathogens | *Copaifera paupera, C. Pubiflora Benth, C. Reticulata Ducke* | Antibacterial and antivirulence | In vitro | Scientific reports | 3 | 3 | 10.1038/s41598-021-84480-7 |
| PAVAN, E; DAMAZO, AS; ARUNACHALAM, K; ALMEIDA, POD; OLIVEIRA, DM; VENTURINI, CL; FIGUEIREDO, FD; DA CRUZ, TCD; DA SILVA, JV; MARTINS, DTD | 2021 | Copaifera malmei harms leaves infusion attenuates tnbs-ulcerative colitis through modulation of cytokines, oxidative stress and mucus in experimental rats | *Copaifera malmei Harms* | Antiulcerative | In vivo | Journal of ethnopharmacology | 3 | 3 | 10.1016/j.jep.2020.113499 |
| PAIXAO, VLB; DE CARVALHO, JF | 2021 | Essential oil therapy in rheumatic diseases: a systematic review | *Copaifera spp* | Anti-inflammatory | Review paper | Complementary therapies in clinical practice | 2 | 2 | 10.1016/j.ctcp.2021.101391 |
| OZELIN, SD; SENEDESE, JM; ALVES, JM; MUNARI, CC; DA COSTA, JD; RESENDE, FA; CAMPOS, DL; LIMA, IMD; ANDRADE, AF; VARANDA, EA; BASTOS, JK; TAVARES, DC | 2021 | Preventive activity of copaifera langsdorffii desf. Leaves extract and its major compounds, afzelin and quercitrin, on dna damage in in vitro and in vivo models | *Copaifera langsdorffii* | Prevent DNA damage | In vitro and in vivo | Journal of toxicology and environmental health-part a-current issues | 2 | 2 | 10.1080/15287394.2021.1898505 |
| KAVALLIERATOS, NG; BOUKOUVALA, MC; NTALAKA, CT; SKOURTI, A; NIKA, EP; MAGGI, F; SPINOZZI, E; MAZZARA, E; PETRELLI, R; LUPIDI, G; GIORDANI, C; BENELLI, G | 2021 | Efficacy of 12 commercial essential oils as wheat protectants against stored-product beetles, and their acetylcholinesterase inhibitory activity | *Copaifera officinalis* | Acetylcholinesterase inhibitory activity | In vitro | Entomologia generalis | 1 | 1 | 10.1127/entomologia/2021/1255 |
| VALADAS, LAR; LOBO, PLD; FONSECA, SGD; FECHINE, FV; NETO, EMR; FONTELES, MMD; TREVIA, LRD; VASCONCELOS, HLP; LIMA, SMD; LOTIF, MAL; FERNANDES, AMB; BANDEIRA, MAM | 2021 | Clinical and antimicrobial evaluation of copaifera langsdorffii desf. Dental varnish in children: a clinical study | *Copaifera langsdorffii Desf* | Antimicrobial | Human reports | Evidence-based complementary and alternative medicine | 1 | 1 | 10.1155/2021/6647849 |
| REIZNAUTT, CM; RIBEIRO, JS; KREPS, E; DA ROSA, WLO; DE LACERDA, H; PERALTA, SL; BOTTINO, MC; LUND, RG | 2021 | Development and properties of endodontic resin sealers with natural oils | *Copaifera spp* | Antimicrobial | In vitro | Journal of dentistry | 1 | 1 | 10.1016/j.jdent.2020.103538 |
| MILHO, C; SILVA, J; GUIMARAES, R; FERREIRA, ICFR; BARROS, L; ALVES, MJ | 2021 | Antimicrobials from medicinal plants: an emergent strategy to control oral biofilms | *Copaifera spp* | Antimicrobial | Review paper | Applied sciences-basel | 1 | 1 | 10.3390/app11094020 |
| ARAUJO, EAM; LIMA, GR; DE MELO, LAD; DE SOUSA, LB; DE VASCONCELLOS, MC; CONDE, NCD; TODA, C; HANAN, SA; ALVES, AD; BANDEIRA, MFCL | 2021 | Effect of a copaiba oil-based dental biomodifier on the inhibition of metalloproteinase in adhesive restoration | *Copaifera multijuga* | Antiproteolytic | In situ | Advances in pharmacological and pharmaceutical sciences | 1 | 1 | 10.1155/2021/8840570 |
| PROPHIRO, JS; DA SILVA, MAN; DE OLIVEIRA, JG; VARIZA, PF; DE LEMOS, AB; PILZ, HL; DA SILVA, OS | 2021 | Change in susceptibility response of aedes aegypti (diptera: culicidae) to organophosphate insecticide and copaifera oleoresin | *Copaifera spp* | Inseticidal | In vitro | Acta tropica | 1 | 1 | 10.1016/j.actatropica.2021.106014 |
| CAMPOS, C; TURCK, P; TAVARES, AMV; CORSSAC, G; LACERDA, D; ARAUJO, A; LLESUY, S; KLEIN, AB | 2021 | Effects of copaiba oil in peripheral markers of oxidative stress in a model of cor pulmonale in rats | *Copaifera spp* | Peripheral Markers of Oxidative Stress | In vivo | Arquivos brasileiros de cardiologia | 1 | 1 | 10.36660/abc.20200929 |
| LEMOS, BJM; SOUZA, FM; ARNHOLD, E; CONCEICAO, EC; COUTO, VRM; FERNANDES, JJR | 2021 | Effects of plant extracts from stryphnodendron adstringens (mart.) Coville, lafoensia pacari a. St.-hil, copaifera spp., and pterodon emarginatus vogel on in vitro rumen fermentation | *Copaifera spp* | Rumen fermentation | In vitro | Journal of animal physiology and animal nutrition | 1 | 1 | 10.1111/jpn.13502 |
| ALOKE, C; EMELIKE, CU; OBASI, NA; OGBU, PN; EDEOGU, CO; UZOMBA, CG; EKAKITIE, O; IYANIWURA, AA; OKORO, CC; OKEY, BP; ANINJOKU, GG; USHAHEMBA, BC | 2021 | Hplc profiling and studies on copaifera salikounda methanol leaf extract on phenylhydrazine-induced hematotoxicity and oxidative stress in rats | *Copaifera salikounda* | Anti-anemic and antioxidant | In vitro and in vivo | Arabian journal of chemistry | 0 | 0 | 10.1016/j.arabjc.2021.103428 |
| ALOKE, C; IGWE, ES; OBASI, NA; AMU, PA; OGBONNIA, EC | 2021 | Anti-diabetic effect of ethanol extract of copaifera salikounda (heckel) against alloxan-induced diabetes in rats | *Copaifera salikounda* | ANTI-DIABETIC and antioxidant | In vivo | Slovenian veterinary research | 0 | 0 | 10.26873/SVR-1072-2020 |
| COUTO, RSD; RODRIGUES, MFSD; FERREIRA, LS; DINIZ, IMA; SILVA, FD; LOPEZ, TCC; LIMA, RR; MARQUES, MM | 2021 | Evaluation of resin-based material containing copaiba oleoresin (copaifera reticulata ducke): biological effects on the human dental pulp stem cells | *Copaifera Reticulata Ducke* | Anti-inflammatory | In vitro | Biomolecules | 0 | 0 | Not informed |
| DE CARVALHO, CR; MAIA, MQ; SOBRAL, M; PEREIRA, GMD; DA SILVA, K; VITAL, MJS; ZILLI, JE; ROSA, CA; ROSA, LH | 2021 | Diversity and antimicrobial activity of culturable endophytic fungi associated with the neotropical ethnomedicinal plants copaifera langsdorffii and copaifera pubiflora | *Copaifera langsdorffii and Copaifera pubiflora* | Antimicrobial | In vitro | South african journal of botany | 0 | 0 | 10.1016/j.sajb.2021.06.021 |
| DOS SANTOS, ACM; OLIVEIRA, VC; MACEDO, AP; BASTOS, JK; OGASAWARA, MS; WATANABE, E; CHAGURI, IM; SILVA-LOVATO, CH; PARANHOS, HFO | 2021 | Effectiveness of oil-based denture dentifrices-organoleptic characteristics, physicochemical properties and antimicrobial action | *Copaifera officinalis* | Antimicrobial | In vitro | Antibiotics-basel | 0 | 0 | 10.3390/antibiotics10070813 |
| DA CRUZ, CBL; SOUSA, LF; LIMA, DA; DE GOIS, JI; DE OLIVEIRA, ED | 2021 | Effect of phonophoresis and copaiba oil on oxidative stress biomarkers after skeletal muscle injury in rats | *Copaifera spp* | Antioxidant | In vivo | Ultrasound in medicine and biology | 0 | 0 | 10.1016/j.ultrasmedbio.2021.04.007 |
| MONTESCHIO, JD; JUNIOR, FMD; DA SILVA, ALA; DAS CHAGAS, RA; FERNANDES, T; LEONARDO, AP; KANEKO, IN; PINTO, LAD; GUERRERO, A; DE MELO, AA; FERRAZ, VP; FAGUNDES, GM; MUIR, JP | 2021 | Effect of copaiba essential oil (copaifera officinalis l.) As a natural preservative on the oxidation and shelf life of sheep burgers | *Copaifera officinalis* | Antioxidant | In vitro | Plos one | 0 | 0 | 10.1371/journal.pone.0248499 |
| ROUMY, V; MACEDO, JCR; BONNEAU, N; SAMAILLIE, J; AZAROUAL, N; ENCINAS, LA; RIVIERE, C; HENNEBELLE, T; SAHPAZ, S; ANTHERIEU, S; PINCON, C; NEUT, C; SIAH, A; GUTIERREZ-CHOQUEVILCA, AL; RUIZ, L | 2021 | Plant therapy in the Peruvian Amazon (Loreto) in case of infectious diseases and its antimicrobial evaluation | *Copaifera paupera* | Antimicrobial | In vitro | Journal of ethnopharmacology | 7 | 7 | 10.1016/j.biopha.2020.111109 |
| WAIBEL, J; PATEL, H; CULL, E; SIDHU, R; LUPATINI, R | 2021 | Prospective, randomized, double-blind, placebo-controlled study on efficacy of copaiba oil in silicone-based gel to reduce scar formation | *Copaifera spp* | Healing | Human reports | Dermatology and therapy | 0 | 0 | 10.1007/s13555-021-00634-5 |
| CAMPANHOLI, KDS; GONCALVES, RS; DA SILVA, JB; DOS SANTOS, RS; DE OLIVEIRA, MC; FERREIRA, SBD; DE CASTRO-HOSHINO, LV; BALBINOT, RB; LAZARIN-BIDOIA, D; BAESSO, ML; BRUSCHI, ML; NAKAMURA, CV; CAETANO, W | 2022 | *Thermal stimuli-responsive topical platform based on copaiba oil-resin: design and performance upon ex-vivo human skin* | *Copaifera reticulata Ducke* | Anti-leishimanial | In vitro | Journal of molecular liquids | 1 | 0 | 10.1016/j.molliq.2022.119625 |
| SIMARO, GV; LEMOS, M; DA SILVA, JJM; CUNHA, WR; CARNEIRO, LJ; AMBROSIO, SR; CUNHA, NL; DE ANDRADE, SF; ARRUDA, C; BANDERO, VC; SASSE, A; SHERIDAN, H; BASTOS, JK; SILVA, MLAE | 2022 | In vivo study of anti-inflammatory and antinociceptive activities of copaifera pubiflora benth oleoresin | *Copaifera pubiflora Benth* | Anti-inflammatory and antinociceptive | In vivo | Natural product research | 0 | 0 | 10.1080/14786419.2020.1855639 |
| CAMPANHOLI, KDS; DA SILVA, RC; GONCALVES, RS; DA SILVA, JB; DE MORAIS, FAP; DOS SANTOS, RS; VILSINSKI, BH; DE OLIVEIRA, GLM; POZZA, MSD; BRUSCHI, ML; SARAIVA, BB; NAKAMURA, CV; CAETANO, W | 2022 | Design and optimization of a natural medicine from copaifera reticulata ducke for skin wound care | *Copaifera reticulata Ducke* | Anti-inflammatory, antimicrobial, healing | In vitro and in vivo | Polymers | 0 | 0 | 10.3390/polym14214483 |
| DOS SANTOS, VRN; MOTTA, JVD; FRAZAO, DR; FERREIRA, RD; SOUZA-MONTEIRO, D; BAIA-DA-SILVA, DC; MENDES, PFS; BITTENCOURT, LO; DE MOURA, JDM; LAMEIRA, OA; BALBINOT, GD; COLLARES, FM; ROSING, CK; LIMA, RR | 2022 | Biological activity of copaiba in damage to the alveolar bone in a model of periodontitis induced in rats | *Copaifera reticulata Ducke* | Anti-inflammatory, antimicrobial | In vivo | Molecules | 0 | 0 | 10.3390/molecules27196255 |
| MALHEIROS, DF; VIDEIRA, MN; FERREIRA, IM; TAVARES-DIAS, M | 2022 | Anthelmintic efficacy of Copaifera reticulata oleoresin in the control of monogeneans and haematological and histopathological effects on Colossoma macropomum | *Copaifera reticulata Ducke* | Anthelmintic | in vitro | AQUACULTURE RESEARCH | 0 | 0 | 10.1111/are.15910 |
| AL-MADBOLY, LA; ABD EL-SALAM, MA; BASTOS, JK; EL-SHORBAGY, SH; EL-MORSI, RM | 2022 | Novel Preclinical Study of Galloylquinic Acid Compounds from Copaifera lucens with Potent Antifungal Activity against Vaginal Candidiasis Induced in a Murine Model via Multitarget Modes of Action | Copaifera lucens | Antifungal | in vitro | MICROBIOLOGY SPECTRUM | 0 | 0 | 10.1128/spectrum.02724-21 |
